# Preclinical evaluation of [^18^F]fluoroethylresorufin as PET tracer for cerebral amyloid angiopathy

**DOI:** 10.1186/s13550-026-01470-4

**Published:** 2026-07-16

**Authors:** Daniel Bleher, Marilena Poxleitner, Ann-Kathrin Grotegerd, Oliver Hihn, Laura Kuebler, Gregory Bowden, Martin Schaller, Florian C. Maier, Bettina Weigelin, Bernd J. Pichler, Andreas Maurer, Kristina Herfert

**Affiliations:** 1 Werner Siemens Imaging Center, Department of Preclinical Imaging and Radiopharmacy, University of Tübingen, Tübingen, Germany; 2 Cluster of Excellence iFIT (EXC 2180) Image Guided and Functionally Instructed Tumor Therapies, University of Tübingen, Tübingen, Germany; 3 Department of Dermatology, University of Tübingen, Tübingen, Germany

**Keywords:** β-amyloid imaging, PET tracer development, Cerebral amyloid angiopathy, Fluorine-18 radiochemistry, Radioligand binding assay, Autoradiography, Transgenic animal imaging, Pharmacokinetics and metabolism

## Abstract

**Background:**

Cerebral amyloid angiopathy (CAA) is characterized by the accumulation of β-amyloid (Aβ) in cerebral vessel walls, predominantly Aβ_1−40_, and frequently co-occurs with Alzheimer’s disease (AD). Reliable in vivo discrimination between vascular and parenchymal Aβ deposits (PEA) in AD is crucial for the assessment of CAA-related risks in anti-Aβ immunotherapies. Current Aβ PET tracers lack the ability to distinguish between vascular and parenchymal Aβ. The phenoxazine derivatives resorufin and ethyl-resorufin were previously shown to bind preferably to CAA over PEA. We therefore evaluated the fluorine-18-labeled resorufin derivative, [^18^F]fluoroethylresorufin ([^18^F]FER) as a potential PET tracer for selective in vivo detection of vascular Aβ.

**Results:**

The binding specificity of [^3^H]FER was assessed using recombinant Aβ_1−40_ and Aβ_1−42_ fibrils as well as in vitro autoradiography on mouse brain tissue of APP23 (CAA+/PEA+) and APPPS1 (CAA-/PEA+) models, and compared to those of [^3^H]PIB. [^18^F]FER and its deuterated analog were synthesized for evaluation of brain pharmacokinetics, metabolism, and in vivo binding using PET imaging in wild-type, APPPS1, and APP23 mice. [^3^H]FER bound with higher affinity to Aβ_1−40_ (K_d_ = 9.4 nM) than to Aβ_1−42_ (K_d_ = 89.1 nM), consistent with its intended CAA selectivity. Autoradiography revealed preferential labeling of vascular amyloid in APP23 mice, with minimal binding in APPPS1 and wild-type tissue. In vivo, [^18^F]FER exhibited high brain uptake (peak SUV = 1.5) and rapid clearance, but metabolic degradation was fast, with ~ 45% parent fraction remaining in the brain at 15 min. Deuteration did not improve stability but did improve washout kinetics. PET imaging with (d_4_)-[^18^F]FER demonstrated a cortical uptake pattern similar to [^11^C]PIB, indicating non-selective binding in vivo.

**Conclusions:**

[^18^F]FER showed promising in vitro affinity for vascular amyloid but lacked in vivo selectivity, likely due to rapid metabolism and high lipophilicity. These findings highlight the challenges of achieving in vivo CAA specificity and provide guidance for the optimization of future tracers targeting vascular Aβ pathology.

**Supplementary Information:**

The online version contains supplementary material available at 10.1186/s13550-026-01470-4.

## Introduction

Cerebral amyloid angiopathy (CAA) is a cerebrovascular disorder characterized by β-amyloid (Aβ) deposition in the walls of small and medium-sized arteries, arterioles, and capillaries of the cerebral cortex and leptomeninges [1]. CAA is observed in up to 80% of Alzheimer’s disease (AD) cases and in approximately 50% of the elderly population [2]. The vascular Aβ deposits in CAA are primarily composed of Aβ_1−40,_ in contrast to the parenchymal Aβ deposits (PEA) in AD, which are enriched in Aβ_1−42_ [3, 4]. These deposits contribute to microvascular degeneration, microhemorrhages, and increased risk of intracerebral hemorrhage, making CAA an important comorbidity in dementia and cerebrovascular disease [2, 5–7]. A recent study has identified prominent concurrent CAA pathology as a pathological substrate of Lewy Body Dementia (LBD) and shown that prominent CAA distinguishes LBD from Parkinson’s disease dementia (PDD), making it a valuable criterion for differential diagnosis [5]. In patients with a genetic predisposition for AD, termed familial Alzheimer’s disease, these processes take place decades before the first clinical symptoms are recognized [8, 9].

Despite its clinical relevance, CAA remains underdiagnosed because definite confirmation still requires post-mortem histopathology according to the modified Boston criteria [6]. Non-invasive diagnostic methods capable of identifying vascular amyloid in vivo are therefore urgently needed.

Positron emission tomography (PET) imaging of Aβ pathology has revolutionized the in vivo assessment of AD [10]. Radiolabeled ligands such as [^11^C]Pittsburgh Compound B ([^11^C]PIB), [^18^F]Florbetapir, [^18^F]Flutemetamol, and [^18^F]Florbetaben, visualize the fibrillar structure of Aβ and enable quantitative analysis of amyloid burden in patients [11]. These tracers have been instrumental, not only for differential diagnosis, but also for the development and monitoring of disease-modifying therapies targeting Aβ aggregation. Thus, a CAA-selective PET tracer would be of great clinical value for differential diagnosis, as current amyloid tracers show overlapping cortical binding patterns between CAA and AD [12]. A tracer with vascular selectivity could delineate the distinct regional distribution of vascular amyloid, particularly the occipital predominance characteristic of CAA, and further support clinical differentiation between CAA, AD, and other proteinopathies, such as LBD [13, 14].

The recent approval of Aβ-targeting antibodies, such as lecanemab and donanemab, marks a milestone in the treatment of AD by demonstrating clinically relevant slowing of cognitive decline through amyloid clearance [15, 16]. However, these novel therapies come with severe side effects, the amyloid-related imaging abnormalities (ARIA), consisting of either edema (ARIA-E) or hemorrhages (ARIA-H), which can lead to treatment interruption or discontinuation [17]. CAA has been found to be a major risk factor for ARIA in Aβ-immunotherapy, underscoring the need for imaging biomarkers that can detect vascular amyloid separately from parenchymal plaques [18–22].

The currently available Aβ PET tracers, lack this selectivity since they bind to the cross-β structure common in both forms of amyloid. Han et al. [23] reported the benzoxazinole derivative resorufin as a potential candidate for vascular Aβ imaging. They reported preferential binding to CAA over neuritic plaques in transgenic mouse models, although chemical stability and poor brain permeability limited its in vivo applicability. Introduction of an ethyl group improved lipophilicity and redox stability, but did not permit fluorine-18 radiolabeling, which is essential for PET imaging. Moreover, subsequent reports described inconsistent selectivity of resorufin analogs, suggesting that their apparent preference for CAA may depend on experimental conditions rather than intrinsic binding specificity [24, 25].

A fluorinated benzoxazinole derivative, fluoroethylresorufin (FER) has been proposed as a potential CAA-selective tracer in a patent application [26]. However, as no peer-reviewed follow-up publication is available, the concept required experimental validation, which we therefore aimed to address in the present study. To this end, we synthesized [^18^F]fluoroethylresorufin ([^18^F]FER), a fluorinated derivative of resorufin with suitable labeling chemistry and improved physicochemical properties. The aim of this study was to systematically evaluate [^18^F]FER and its deuterated analog (d_4_)-[^18^F]FER as potential PET tracers for vascular Aβ imaging. We characterized their binding affinities for Aβ_1−40_ and Aβ_1−42_ fibrils, assessed their binding patterns in brain tissue from transgenic mouse models of β-amyloidosis with distinct Aβ isoform profiles, and compared their in vivo pharmacokinetic and metabolic behavior to that of the established tracer [^11^C]PIB. Through this multilevel preclinical evaluation, we sought to determine whether the resorufin scaffold can provide the necessary selectivity and stability for reliable in vivo detection of CAA.

## Materials and methods

### Chemistry

**Fig. 1:**
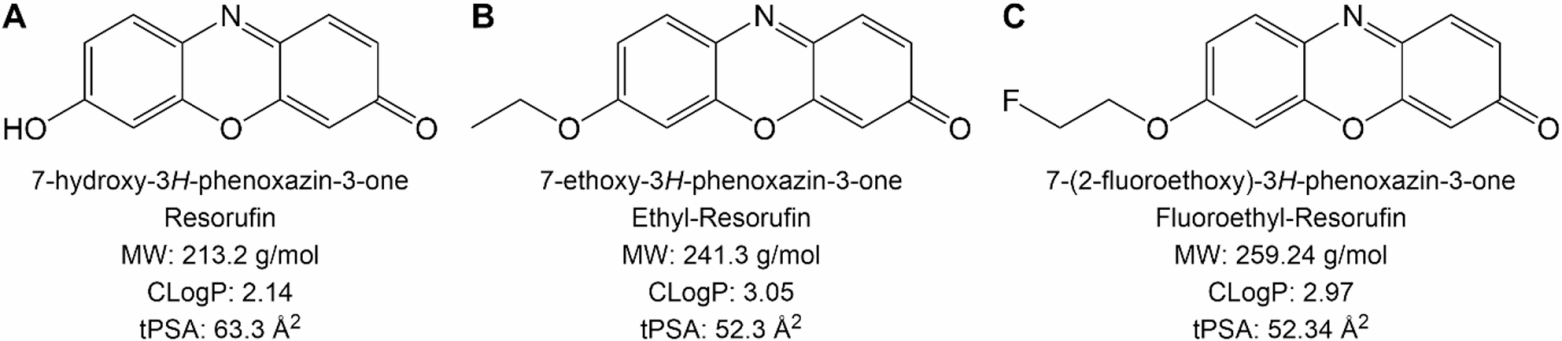
Chemical structures and properties of resorufin analogs. Chemical structures of (**A**) resorufin, (**B**) ethylresorufin, and (**C**) fluoroethylresorufin (FER). For each compound molecular weight (MW), calculated logP (clogP), and topological polar surface area (tPSA) are indicated

The precursor and non-radioactive standard fluoroethylresorufin were synthesized as previously reported [26], a synthesis description is shown in the supplementary information. The chemical structures are shown in Figure S2. The calculated logP (clogP) and topological polar surface area (tPSA, [Å^2^]) values shown in Fig. 1 were calculated by the Molinspiration property engine (v2021.10). The fluorescent reference dye ICTAD-1 (imino-coumarin-thiazole for AD) was synthesized, adapted from the previously described synthesis, and is described in the supplementary information [27].

### Aggregation and characterization of Aβ fibrils

Aβ_1−40_ and Aβ_1−42_ fibril production was adapted with several changes [28–31]. Briefly, synthetic lyophilized human Aβ_1−40_ (EMC microcollections GmbH, Tübingen, Germany) was dissolved in 50 mM sodium phosphate buffer (pH 7.4) with 0.02% sodium azide for a final monomer concentration of 250 µM. Synthetic lyophilized human Aβ_1−42_ (Intavis peptide services GmbH, Tübingen, Germany) was dissolved in 25 mM Tris*HCl (pH 7.6) with 5% DMSO and 0.02% sodium azide for a final monomer concentration of 250 µM. Both Aβ_1−40_ and Aβ_1−42_ monomers were then aggregated at 800 rpm for 96 h at 37 °C in a Thermoshaker (Eppendorf SE, Hamburg, Germany). After aggregation, fibrils were sonicated for 3 min in a bath sonicator, aliquoted, and snap-frozen in liquid nitrogen.

Aβ fibril characterization measured via Thioflavin-T (ThT) fluorescence spectroscopy and negative staining transmission electron microscopy (TEM), as described previously [32]. For TEM, fibrils were placed directly onto a glow-discharged electron microscopy grid, and after adsorption, the grid was washed with H_2_O and stained with 1% UO_2_(CH_3_COO)_2_. Images were generated using a Zeiss LIBRA 120 TEM (Carl Zeiss, Oberkochen, Germany) operating at 120 kV. Fibril lengths were measured with the software Fiji (ImageJ, Version 1.54f, NIH, USA) and further analyzed with GraphPad Prism (GraphPad Prism version 6.04 for Windows, GraphPad Software, www.graphpad.com).

### Fluorescence and UV/VIS spectroscopy

**Fig. 2:**
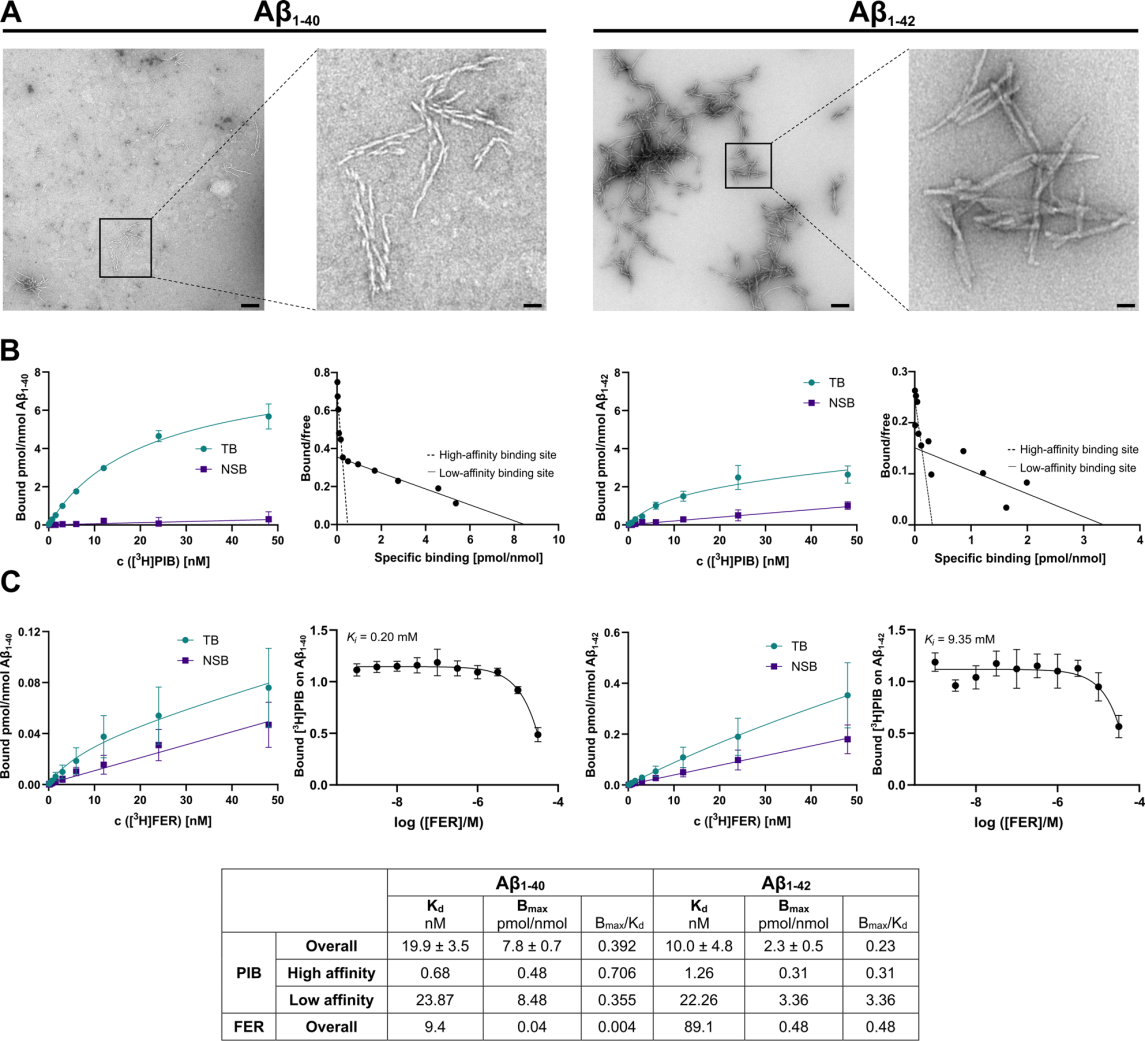
Binding specificity and selectivity of FER on Aβ_1−40_ and Aβ_1−42_ fibrils and comparison to [^3^H]PIB. (**A**) Representative negative-stain transmission electron microscopy (TEM) images of Aβ_1−40_ (left) and Aβ_1−42_ (right) fibrils used in binding assays. (**B**) Saturation binding curves and Scatchard analysis of [^3^H]PIB to Aβ_1−40_ (left), and Aβ_1−42_ (right) fibrils, showing two distinct binding sites. (**C**) [^3^H]FER binding curves and competition of [^3^H]PIB binding with increasing concentrations of FER for Aβ_1−40_ (left) and Aβ_1−42_ (right) fibrils, demonstrating distinct binding sites and isoform preference. Abbrevations: PIB, Pittsburgh Compound B; FER, fluoroethylresorufin, TB, total binding, NSB, non-specific binding. Scale Bar 100 nm (20 nm in the zoomed image)

FER absorption spectra were measured in phosphate buffered saline (PBS) (pH 7.4) with 25% DMSO in a scan range of 300 to 600 nm in a Lambda 25 UV/VIS spectrometer (PerkinElmer, Waltham, MA, USA). The resulting data were transferred to the UVWinLab data processor (PerkinElmer) and then plotted in GraphPad Prism. Fluorescence spectra were obtained in the same cuvette under identical buffer conditions. Fluorescence spectroscopy was performed with a LS42 fluorescence spectrometer (PerkinElmer) in a quartz cuvette. Spectra were recorded with FL WinLab 2.0 (PerkinElmer), exported, and transferred to GraphPad Prism 9.4. FER (fluoroethyl-resorufin; 7-(2-fluoroethoxy)-3*H*-phenoxazin-3-one) revealed one single absorbance maximum at $\:{\uplambda\:}$_max, abs_ = 477 nm (Fig. S1, A) and a linear absorbance range with ε = 1.17*10^− 4^ L*mol^− 1^*cm^− 1^ (Fig. S1, D). When excited at $\:{\uplambda\:}$_ex_ = 477 nm, FER produces one single fluorescence emission maximum at 577 nm (Fig. S1, B). The presence of β-helical secondary structure in amyloid fibrils was confirmed via ThT fluorescence emission. Briefly, an emission spectrum of 9.6 µl Aβ fibrils (250 µM) diluted in 50 mM glycine NaOH pH 8.5 with 10 µM ThT to a final Aβ concentration of 800 nM, incubated in the dark at room temperature for 30 min, was carried out, with an excitation wavelength of 450 nm, as described previously [32]. Aβ_1−40_ and Aβ_1−42_ fibrils show increased ThT fluorescence emission (Fig. S1, F and G), and characteristic fibril morphology with negative-staining TEM (Fig. 2, A), revealing mean lengths of 110 ± 75 nm for Aβ_1−40_ and 148 ± 84 nm for Aβ_1−42_ (Fig. S1, H and I). Addition of 500 nM Aβ_1−40_ fibrils to FER did not change fluorescence emission (Fig. S1, C and E).

### FER and PIB tritiation

[^3^H]FER and [^3^H]PIB were produced and purified by RC Tritec AG (Teufen, Switzerland); their molar activity was determined by mass spectrometry (MS) using an iterative residue correction method, and their radiochemical purity was determined via high performance liquid chromatography (HPLC). FER was tritiated by specific labeling of the fluoroethyl residue to give a molar activity of 1.80 GBq/µmol (48.6 Ci/mmol) with an isotopic abundance of ^1^H-4.6%; ^3^H_1_-22.7%; ^3^H_2_-72.6% and a radiochemical purity (RCP) of 96.9%. PIB was tritiated by unspecific tritiation of the unlabeled reference compound (6-OH-BTA-1) to give a molar activity of 0.8 GBq/µmol (22.1 Ci/mmol) and an RCP of > 99%. Compounds were stored at -80 °C until further usage.

### Fibril binding assays

Saturation binding experiments were performed to evaluate the binding specificity towards Aβ_1−40_ and Aβ_1−42_. Binding experiments were conducted as previously described [29–31]. Briefly, for competition binding assays, a fixed concentration of Aβ_1−40_ or Aβ_1−42_ (500 nM) fibrils were incubated with 10 nM [^3^H]PIB in low-binding plates (96-well micro test plate, Ratiolab GmbH, Dreieich, Germany). Competition was carried out by adding decreasing concentration of a 1:√10 dilution series of FER (10 µM – 1 nM) in 30 mM Tris*HCl, pH 7.4, with 10% EtOH and 0.1% bovine serum albumin (BSA). For saturation binding assays, 1:2 serial dilutions of [^3^H]PIB and [^3^H]FER ranging from 48 nM to 23 pM were prepared in buffer (50 mM Tris/HCl buffer (pH = 7.4) with 10% EtOH, 0.1% BSA and 0.025% NaN_3_) and added to 500 nM (for [^3^H]PIB) for 2.5 µM (for [^3^H]FER) fibrils of either Aβ_1−40_ or Aβ_1−42_. To determine total binding (TB), 150 µL of [^3^H]PIB or [^3^H]FER dilutions were added to the fibrils. Non-specific binding (NSB) was determined by adding the unlabeled compounds in excess (2.5 µM final concentration of PIB and 400 nM of FER) to the tracer solution in a second set of binding reactions. Competition and saturation assays were performed in a total volume of 200 µl/well.

Incubation was performed at 45 rpm at 37 °C for two hours on a shaker while covered by removable sealing tape (Revvity Diagnostics GmbH, Lübeck, Germany) to prevent evaporation. After incubation, bound and free tracers were separated by vacuum filtration using a filtermat harvester (Revvity Diagnostics GmbH), and fibrils were filtered onto a glass fiber filtermat type B (Revvity Diagnostics GmbH) pre-incubated with 1% polyethyleneimine for 30 min at 4 °C. The filtermat was washed twice with 100 mL of the respective incubation buffer, and dried in a microwave. Melt-on scintillator sheets (MeltiLex™ B/HS, Revvity Diagnostics GmbH) were molten into the filtermat at 120 °C, and after solidification at room temperature, the filter was sealed in a MicroBeta sample bag (Revvity Diagnostics GmbH) and measured using the Wallac MicroBeta^®^ TriLux scintillation counter (Revvity Diagnostics GmbH). Radioactivity converted to bound pmol ligand per added nmol of total fibril was plotted against increasing concentration in log(M) (for competition assay) or in nM (for saturation assay). Data points were fitted using non-linear regression analysis in GraphPad Prism.

### Radiosynthesis of [^18^F]FER, (d_4_)-[^18^F]FER, and [^11^C]PIB

Radiolabeling of [^18^F]FER (7-(2-(fluoro-^18^*F*)ethoxy)-3*H*-phenoxazin-3-one) and (d_4_)-[^18^F]FER (7-(2-(fluoro-^18^*F*)ethoxy-1,1,2,2-*d*_4_)-3*H*-phenoxazin-3-one) was carried out by automated radiolabeling of the tosylate precursor with subsequent purification. [^18^F]Fluoride was produced using a PETtrace 890 cyclotron (GE Healthcare GmbH, Uppsala, Sweden) using the ^18^O(p, n)^18^F nuclear reaction by proton bombardment of [^18^O]H_2_O, and delivered into a modified GE Tracerlab module (GE Healthcare GmbH). It was trapped onto a preconditioned quaternary methyl ammonium (QMA) cartridge, eluted into the reactor with a solution of 9.5 mg Kryptofix, 1.7 mg K_2_CO_3_, 80 µl H_2_O, and 1.92 ml MeCN, and the solvent was evaporated at 90 °C. After cooling, 2 mg of the tosylate precursor 2-((3-oxo-3*H*-phenoxazin-7-yl)oxy)ethyl-4-methylbenzenesulfonate or 2-((3-oxo-3*H*-phenoxazin-7-yl)oxy)ethyl-1,1,2,2-*d*_*4*_-4-methylbenzenesulfonate dissolved in 500 µl dimethylformamide (DMF) was added, and the reaction mixture was heated to 70 °C for 10 min. The resulting mixture was diluted with 1.5 mL of H_2_O, and the mixture was injected into the HPLC loop. Semipreparative HPLC conditions for purification: Luna 10 μm C18 (2) 100 Å 250 mm × 10 mm (Phenomenex, Torrance, CA, USA); 45% MeCN in 0.1% TFA in H_2_O (retention time ≈ 8 min); 5 ml/min. After successful peak cutting, the product was diluted with 60 mL of H_2_O and trapped onto a C18 cartridge that was pre-conditioned with 10 mL of EtOH and 10 mL of H_2_O. After elution with 0.5 mL DMSO, the product was formulated with 5 mL of a 1:3 mixture of polyethylene glycol (PEG400) and PBS, and leftover fluoride was removed with an Sep-Pak Alumina N Plus Light cartridge (280 mg) before the transfer into the product vial. Quality control was performed by analytical radio-HPLC to determine the (radio-)chemical purity of the radiolabeled compound and the carrier content for the calculation of the molar activity (A_m_). The characterization of [^18^F]FER and (d_4_)-[^18^F]FER are provided in the supplementary information. At the end of the synthesis (EOS), the radiochemical yield (RCY) of [^18^F]FER was 1.85 ± 0.49% (decay-corrected), the molar activity (A_m_) 251 ± 108 GBq/µmol, and the carrier content 0.22 ± 0.11 µg/mL (*n* = 9). The RCY of (d_4_)-[^18^F]FER was 5.25 ± 1.97%, the A_m_ 229 ± 119 GBq/µmol, and the carrier content 0.52 ± 0.28 µg/mL (*n* = 11). The radiochemical purity of [^18^F]FER and of (d_4_)-[^18^F]FER exceeded 98%, with exemplary radio-HPLC chromatograms shown in Figure S2, C.

[^11^C]PIB was routinely produced by the department of Radiopharmacy at the University Hospital Tübingen. The radiosynthesis was performed as previously published [33].

### Animal models

Animals were housed in our vivarium with litter mates of the same sex with up to five mice per cage, consisting of individually ventilated cages with standard-diet food and tap water ad libitum on a 12:12 h light-dark cycle, and were kept at a temperature of 22 °C with 40–60% humidity. Animals were bred at the animal facility of the University Hospital Tübingen (Germany).

Two transgenic mouse models, the APPPS1-21 and the APP23 model, as well as age-matched controls with a C57BL/6 background, were used in the study. The double transgenic APPPS1-21 mice (B6.Cg-Tg(Thy1-APPSw, Thy1- PSEN1*L166P)21Jckr; APPPS1) co-express the human Swedish double mutation APP KM670/671NL and the L166P mutated human PS1 under the control of neuron-specific Thy-1 promoter. This model shows accelerated amyloid deposits at six weeks of age, with Aβ_1−42_ dominating over Aβ_1−40_ with a total Aβ_1−40_/Aβ_1−42_ ratio of 1:5 reported in 8-month old amyloid-depositing mice, and extensive PEA but minimal CAA [34]. For 16–22-month-old APPPS1 mice the total Aβ_1−40_/Aβ_1−42_ was reported as 0.3:1 [35].

The single-transgenic APP23 mice (B6.Cg-Tg(Thy1-APP)3Somm/J) express the human Swedish double mutation APP KM670/671NL under the control of neuron-specific Thy-1 promotor, leading to an extensive PEA, but also to the development of CAA with microhemorrhages, and therefore serves as a tool for the development of diagnostic and therapeutic strategies targeting CAA and related complications [36, 37]. In contrast to APPPS1 mice, APP23 mice show an Aβ ratio of the opposite direction, with Aβ_1−40_ dominating over Aβ_1−42_ with a a total Aβ ratio of 5:1 in 27–28 month-old APP23 mice [35].

Both male and female mice were investigated in the transgenic and control groups. APPPS1 breeding pairs were kindly provided by Prof. Mathias Jucker, and APP23 breeding pairs were purchased from the Jackson Laboratory (Charles River Germany, Sulzfeld, Germany).

At the time of PET experiments APP23 mice were 20.2 ± 0.5 months old, and APPPS1 mice were 15.3 ± 0.6 months old. The selected age windows were based on the literature-reported Aβ_1−40_/Aβ_1−42_ ratios described above and previous longitudinal PET-MRI and histological data indicating a robust amyloid burden in APP23 mice, and plateaued [^11^C]PIB binding and cortical amyloid area in APPPS1 mice at these ages [38]. Animals were therefore selected within an age range that allowed comparability between models while minimizing age-related confounding and preserving the characteristic differences in Aβ composition, while avoiding excessively advanced pathology. The same age ranges were used for in vitro autoradiography and histology experiments.

### Tissue preparation

Mice were sacrificed through asphyxiation with CO_2_, and the brain was removed and prepared for ex vivo analysis by undergoing the following paraffin embedding workflow: Fixation in 4% formalin for 24 h, 70% EtOH, 80% EtOH, 95% EtOH, 100% EtOH, Xylene, paraffin wax (60 °C) and embedding in paraffin blocks with subsequent coronal sectioning using a microtome (Microm HS355S Thermo Fischer Scientific, Darmstadt, Germany) with a thickness of 4 μm. Sections were deparaffinized using the following workflow: Xylene (9 min), 100% EtOH (9 min), 90% EtOH (9 min), 80% EtOH (6 min), 70% EtOH (6 min), 50% EtOH (6 min), H_2_O (3 min), and autoradiography and/or histology was subsequently performed on the respective tissue sections.

### In vitro autoradiography

Autoradiography was performed using [^3^H]FER, (d_4_)-[^18^F]FER, [^11^C]PIB, and [^3^H]PIB. Tissue sections were pre-incubated in autoradiography buffer with the following compositions: for (d_4_)-[^18^F]FER, PBS (pH 7.4) containing 2% of each PEG-400, DMSO, and EtOH; for [^3^H]FER the same buffer composition as for (d_4_)-[^18^F]FER with additional 0.25% Triton-X 100, for [^11^C]PIB, PBS (pH 7.4) containing 10% EtOH; and for [^3^H]PIB the same buffer composition as [^11^C]PIB with additional 0.05% Tween-80). Pre-incubation times were 1 h for (d_4_)-[^18^F]FER, 40 min for [^11^C]PIB, and 20 min for the tritiated ligands.

For PIB autoradiography, total binding was determined by incubation with radiolabeled ligand (3 nM for [^11^C]PIB and 10 nM for [^3^H]PIB), while non-specific binding was assessed by co-incubation with 3 µM of the corresponding unlabeled compound.

For FER autoradiography, non-specific binding was determined by pre-incubating the tissue sections for 10 min with 3 µM unlabeled FER (blocking solution) prior to the addition of the radiolabeled tracer. The blocking solution remained present during subsequent incubation with the radiolabeled ligand (3 nM). Total binding was assessed by incubation with radiolabeled ligand alone.

The sections were washed in autoradiography buffer, PBS and H_2_O for 5 min each, dried, and a storage phosphor plate (Molecular Dynamics Screen, Caesera, Israel for short-living radioisotopes and BAS-IP TR 2040 E, Cytiva, Marlborough, MA, USA for tritium) was exposed to the tissue sections. Exposure times for carbon-11, fluorine-18, and tritium were 3.5 h, 20 h, and 7 days, respectively. The phosphor plates were then scanned with a Storm 840 Phosphorimager (Molecular Dynamics) and processed with the software Fiji (ImageJ, Version 1.54f).

### Ex vivo tissue staining

All staining procedures and solution preparation with light-sensitive dyes were performed without light exposure, using aluminum foil-wrapped materials and a darkened staining chamber. All staining solutions were filtered through a 0.22 μm filter.

Hematoxylin and Eosin (H&E) staining was performed using a standard staining procedure [39].

#### Aβ_1−42_ immunofluorescence (IF)

Aβ_1−42_ IF was adapted from our previously described workflow [31]. Briefly, antigen retrieval was performed on rehydrated sections by incubation in heated sodium citrate (10 mM, pH 6.0) for 30 min. Blocking was carried out in 50 mM Tris-buffered saline with 0.1% Triton-X 100 and 1% bovine serum albumin (TBS-X) with 5% normal goat serum (NGS) added. They were then incubated with rabbit anti-Aβ_1−42_ primary antibody (Cat. No. 218703; Synaptic Systems GmbH, Göttingen, Germany) at a 1:100 dilution in TBS-X overnight at 4 °C. The samples were then washed for 3 × 10 min in TBS-X, and incubated with a goat anti-rabbit Alexa Fluor 568 secondary antibody (Thermo Fischer Scientific, Darmstadt, Germany) diluted 1:250 in TBS-X for 1 h at room temperature in the dark. Sections were then washed 2 × 10 min in TBS-X and 10 min in Tris-buffered saline (TBS), and subsequently mounted with ProLong Glass Antifade Mountant (Thermo Fischer Scientific). All steps involving the secondary antibody were performed in the dark.

PIB staining was performed by incubating the slides in 0.5% PIB dissolved in 100% EtOH (w/v) without previous filtration and subsequently in 0.1% Sudan Black B (previously dissolved in 70% EtOH at 70 °C) to block autofluorescence [40, 41]. After rehydration, the slides were incubated in 100% EtOH for 20 min and afterwards in the PIB staining solution for 5 min with subsequent washing in 70% EtOH for 1 min and incubated in the Sudan Black B solution for 5 min, followed by dipping the slides three times in 70% EtOH.

#### FER staining

The FER staining procedure was adapted from the previously published resorufin staining protocol, considering the different tissue preparation and insolubility of the dye [23]. The staining solution was prepared by dissolving FER to a concentration of 10 µM in PBS-T (PBS with 0.25% TritonX-100) at pH 7.4 with 2% of PEG-400, DMSO, and EtOH. After rehydration, the slides were pre-incubated in staining buffer without FER at room temperature for 30 min and subsequently in the staining solution for 60 min. The slides were washed three times with PBS and once with 50% ethanol in PBS for 5 min each.

#### ICTAD-1 staining

ICTAD-1 is a conformation-sensitive iminocoumarin–thiazole probe that exhibits differential fluorescence emission in the presence of Aβ_1−40_ and Aβ_1−42_ fibrils, thereby enabling their differentiation and spectral-based quantification on tissue slices [27]. The dye was synthesized as previously described and staining was performed with slight modifications of the previously published protocol [27]. The dye was dissolved in acetonitrile (ACN), and this solution was added to a buffer of PBS pH 7.4 with 20% EtOH to give a final ICTAD-1 concentration of 25 µM with 2% of ACN. The staining solution was sonicated in a bath sonicator. Sections were stained for 20 min and washed twice in 50% EtOH in PBS and twice in H_2_O for 1 min each.

After staining, the tissue sections were mounted with Eukitt^®^ quick-hardening mounting medium and stored at room temperature in the absence of light exposure. Fluorescence microscopy was either performed with an Axiovert 200 microscope equipped with an HBO 100 fluorescence lamp, a LD A-Plan 20x/0.30 PH1 objective, and an Axiocam MRC using the Axiovision software Version 4.8 (Carl Zeiss, Oberkochen, Germany) or using a Leica DMi8 microscope interfaced with Leica LAS X software at 10x magnification (Leica Microsystems CMS GmbH, Wetzlar, Germany).

**Fig. 3:**
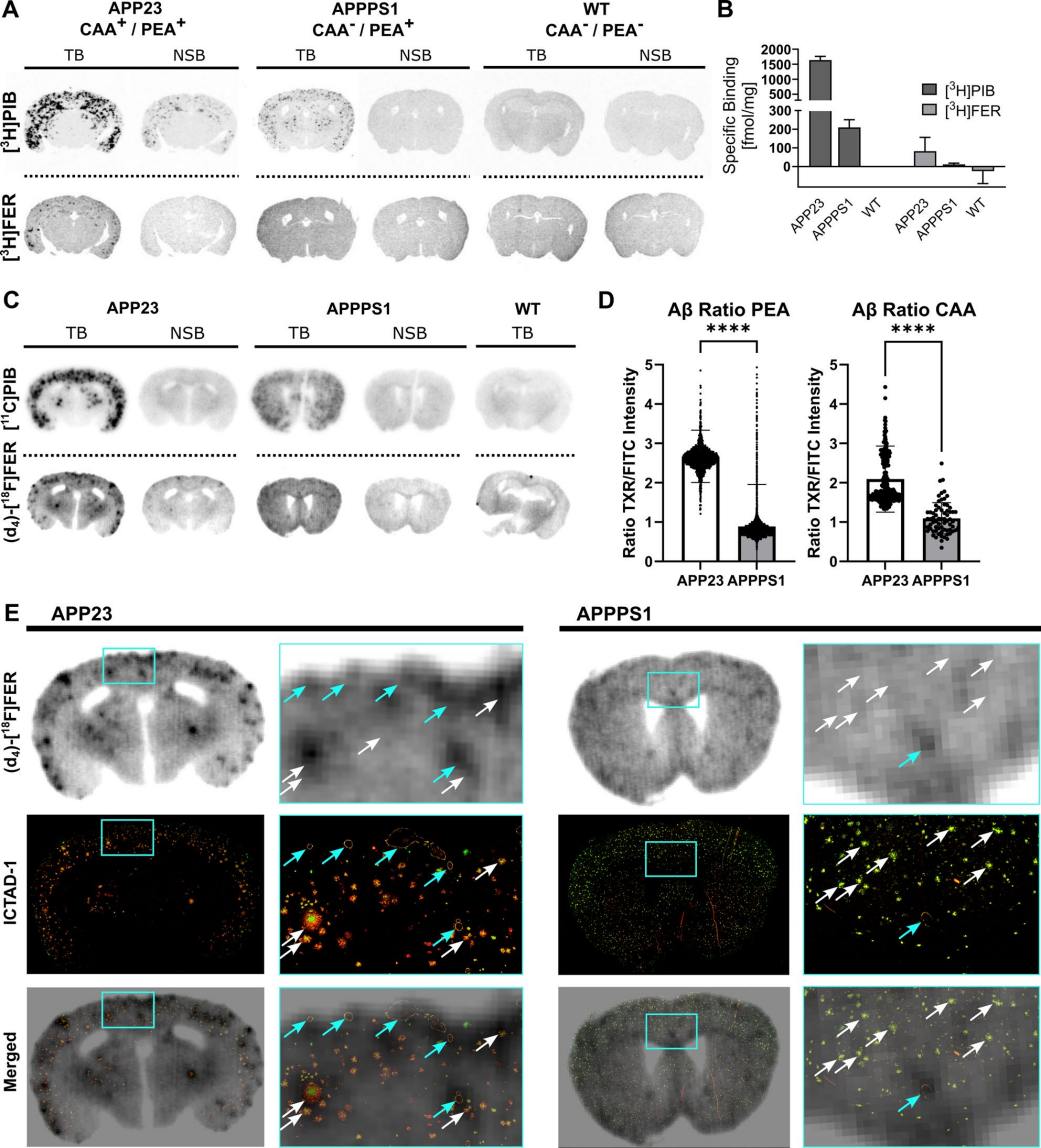
In vitro autoradiography of FER and comparison to PIB on transgenic mouse brain tissue. (**A**) Autoradiographic images showing total (TB) and non-specific binding (NSB) of [^3^H]FER (top) and [^3^H]PIB (bottom) on APP23 (CAA^+^/PEA^+^), APPPS1 (CAA^−^/PEA^+^), and wild-type (CAA^−^/PEA^−^) mouse brain tissue. (**B**) Quantification of specific binding for [^3^H]FER and [^3^H]PIB across genotypes. (**C**) Autoradiography of (d_4_)-[^18^F]FER (top) and [^11^C]PIB (bottom) on APP23 and APPPS1, and wild-type mouse brain sections. (**D**) Quantification of the Aβ_1−40_/Aβ_1−42_ ratio in PEA and CAA in 20.8-month-old APP23 and 15.8-month-old APPPS1 mice (*n* = 2). Each dot represents one Aβ plaque or vessel. (**E**) Colocalization of (d_4_)-[^18^F]FER and ICTAD-1 fluorescence on consecutive sections showing Aβ_1−40_ (red) and Aβ_1−42_ (green) distribution (5x magnification) on cortical regions. White arrows, PEA; blue arrows, CAA. Abbreviations: CAA, cerebral amyloid angiopathy; PEA, parenchymal amyloidosis. PIB, Pittsburgh Compound B; FER, fluoroethylresorufin; WT, wild-type; TB, total binding; NSB, non-specific binding

Three different filter sets were used: 4′,6-Diamidin-2-phenylindol (DAPI; ex: 418 nm/em: 465 nm (blue)), fluorescein isothiocyanate (FITC; ex: 495 nm/em: 525 nm (green)), and tetramethylrhodamine isothiocyanate (TRITC; ex: 555 nm/em: 580 nm (red)). Acquisition of ICTAD-1 stained tissues at the Leica DMi8 was performed with the following filter sets: FITC (ex: 460–500 nm, em: 512–542 nm) and TXR (ex: 540–580 nm, em: 662–738 nm). For proof-of-concept staining of ICTAD-1 (Fig. S4, B), spectral unmixing was applied to correct for spectral bleedthrough between the two emission channels, but was not applied before merging the channels in Fig. 3, C. Calculations of Aβ_1−40_/Aβ_1−42_ ratios were based on intensity threshold–segmented Aβ_1−40_ and Aβ_1−42_ signals measured in the respective channels. Segmentation and measurements were performed using FIJI (version 1.54p). Fluorescence microscopy images were converted to 8-bit RGB format and automated thresholding was applied using the Moments algorithm to separate plaque/vessel signal from the background. The resulting binary masks were used to segment plaque-positive and vessel-positive regions. Quantitative analysis was performed in FIJI by measuring the Integrated Density of the corresponding masks in each channel for every experimental model.

### Ex vivo metabolite analysis in blood plasma and brain

Metabolite analysis was performed as described previously [29, 31]. After i.v. injection of 113 MBq and 110 MBq [^18^F]FER; and 146 MBq and 150 MBq (d_4_)-[^18^F]FER (each for the 5 min and 15 min timepoint, respectively) in 3-month-old C57BL/6 mice, blood samples were collected by heart puncture and centrifuged at 17.000 x g for two minutes at 4 °C. Mice were transcardially perfused with 20 ml of ice-cold PBS. The brain was then removed, transferred to a dounce tissue grinder (Sigma Aldrich GmbH) with 500 µl of ice-cold PBS, and sequentially homogenized using large and small clearance pestles. Both the plasma and the brain homogenate were transferred to a 1.5 ml Eppendorf reaction tube and stored on ice until further analysis. Plasma and brain homogenates were mixed 1:1 with acetonitrile, briefly centrifuged, incubated for 2 min on ice, and precipitated proteins were removed by centrifugation at room temperature for 1.5 min at 12,000 x g (MiniSpin^®^, Eppendorf AG). The supernatant was analyzed by a reversed-phase HPLC on a Luna Phenyl Hexyl column (5 μm, 100 Å, 300 mm x 4.6 mm, Phenomenex) equipped with a radioactivity detector with an isocratic flow rate of 1.5 mL/min (48% MeCN in H_2_O with 0.1% TFA). The radioactivity signal was plotted against time using GraphPad Prism without decay correction. The area under the curve was calculated in % using manual analysis by subtracting the mean background signal, summing up peak intensities, and calculating their ratios so that the peaks add up to 100%. The injected dose was less than 0.5 nmol, and the injected volume did not exceed 5mL/kg.

### In vivo PET and MRI imaging, data acquisition, and analysis

 In vivo experiments were conducted as previously described [31, 32, 39]. At the time of measurement, APP23 mice were 20.2 ± 0.5 months old (*n* = 5; 4 females, 1 male), and APPPS1 mice were 15.2 ± 0.6 months old (*n* = 5; 2 females, 3 males). Controls had a mean age of 17 ± 2.8 months (*n* = 6; 1 female, 5 males). Pharmacokinetic experiments were performed on 3-month-old C57BL/6 mice. PET imaging was performed on three small animal Inveon PET scanners (Siemens Healthcare, Knoxville, TN, USA). Mice were anesthetized with 1.5–1.7% isoflurane evaporated in 100% medical oxygen at a flow rate of 0.8 L/min during the whole measurement. A tail vein catheter was placed, and the animals were positioned head-to-head in the center of the field of view (FOV) on dedicated MR-compatible mouse beds (Medres, Cologne, Germany) and connected to a feedback temperature control unit set to 37 °C. Mice were injected intravenously (i.v.) with either 12.72 ± 0.48 MBq [^18^F]FER or (d_4_)-[^18^F]FER. 9 ± 2 days before the FER scan, transgenic mice as well as the age-matched control group received a prior scan with 11.77 ± 0.75 MBq [^11^C]PIB. Dynamic PET data were acquired for 60 min and divided into 23 frames (8 × 30 s, 6 × 60 s, 7 × 300 s, and 2 × 450 s). For attenuation correction, a transmission measurement using a cobalt-57 point source was performed for an additional 13 min. The data was reconstructed into a dynamic PET image using a OSEM2D with an image zoom of 2 and a 256 × 256 matrix size, resulting in a voxel size of 0.2 × 0.2 × 0.8 mm^3^ (Inveon Acquisition Workplace, Siemens Healthcare, USA), and further analyzed via PMOD software v4.2 (PMOD technologies, Zürich, Switzerland).

Magnetic resonance imaging (MRI) scans were performed subsequently either after the FER or a PIB PET scan. Anatomical MR brain images were acquired on a 7T small animal MRI scanner (Bruker BioSpin, Ettlingen, Germany) with identical anesthesia and temperature conditions. A rat whole-body coil (Bruker BioSpin GmbH) was used for mouse MR measurements. After the mouse was positioned in the center of the FOV, a Turbo Rare 3D T2 sequence 3D T2 sequence (TR = 800 ms; TE = 37.63 ms; FOV = 74 × 32 × 18; image size = 296 × 128 × 72) was used to acquire the anatomical image of the brain.

To account for inter-individual and age-related differences in brain size and potential atrophy, each brain MR dataset was individually adjusted to its native dimensions prior to analysis. For this purpose, MR Images were coregistered to the T2 mouse brain atlas provided by PMOD [42, 43] by scaling the X, Y, and Z dimensions accordingly. Dynamic PET image dimensions were adjusted to the respective MR image dimensions and subsequently coregistered to the MR image. Volumes of interest (VOIs) of different brain regions were extracted, and tissue time activity curves (TACs) were calculated and expressed as standardized uptake values (SUVs), which were calculated as SUV(t) = radioactivity concentration (kBq/mL of the organ)/(injected dose [kBq]/body weight [g]). Half-life (washout) was determined by fitting the data points starting from the peak with a one-phase decay fit in Graphpad. SUV ratios (SUVRs) were calculated as the ratio of the SUV in the target region (cortex) to the SUV in the reference region. The cerebellum was chosen as a reference region due to a lack of pathology and no expected specific uptake. Kinetic modeling was performed in PMOD using the simplified reference tissue model (SRTM) with the cerebellum as reference input to calculate binding potential (BP) values. For averaged SUVR images, mean SUV images from 15 to 40 min were calculated from the coregistered scans and scaled by dividing each voxel by the mean cerebellum value. Single SUVR images for each group were subsequently merged to give the averaged SUVR image.

## Results

### In vitro binding assays

To test the hypothesis that the reported CAA selectivity of resorufin derivatives may be driven by preferential binding to Aβ_1−40_ over Aβ_1−42_, saturation binding assays were performed using synthetic Aβ fibrils. Saturation binding assays with [^3^H]PIB revealed high-affinity binding to Aβ_1−40_ and Aβ_1−42_ (K_d_ = 19.9 ± 3.5 nM for Aβ_1−40_ and 10.0 ± 4.8 nM for Aβ_1−42_) and a high binding capacity (B_max_ = 7.8 ± 0.7 pmol/nmol for Aβ_1−40_ and 2.3 ± 0.5 pmol/nmol for Aβ_1−42_) with a one binding site fit. Scatchard analysis revealed two binding sites for both Aβ_1−40_ and Aβ_1−42_, with very high affinities of 0.68 nM for Aβ_1−40_, with B_max_ = 0.48 pmol/nmol; and 1.26 nM for Aβ_1−42_, with B_max_ = 0.31 pmol/nmol for the high-affinity binding site. The low-affinity binding site revealed affinities of 23.87 nM for Aβ_1−40_, with B_max_ = 8.48 pmol/nmol; and 22.26 nM for Aβ_1−42,_ with B_max_ = 3.36 pmol/nmol (Fig. 2, B). The in vitro B_max_/K_d_ ratios for Aβ_1−40_ were 0.71 mL/nmol for the high affinity binding site, 0.36 mL/nmol for the low affinity binding site, and 0.39 mL/nmol for the one binding site fit. For Aβ_1−42_, the B_max_/K_d_ were 0.25 mL/nmol for the high-affinity binding site, 0.15 mL/nmol for the low-affinity binding site, and 0.23 mL/nmol for the one binding site fit.

[^3^H]FER exhibited nanomolar affinity to both Aβ isoforms, with a 10-fold preference to Aβ_1−40_ (K_d_ = 9.4 nM) over Aβ_1−42_ (K_d_ = 89.1 nM). B_max_ values were 0.04 pmol/nmol for Aβ_1−40_ and 0.48 pmol/nmol for Aβ_1−42_, indicating a smaller number of high-affinity binding sites on Aβ_1−40_, but overall stronger interaction (Fig. 2, C). The B_max_/K_d_ ratios were in the same range (0.004 mL/nmol for Aβ_1−40_ and 0.005 mL/nmol for Aβ_1−42_). Competition studies confirmed that FER binds to a site distinct from that of PIB, as no displacement of [^3^H]PIB binding was observed.

### In vitro evaluation of mouse brain tissue

APP23 and APPPS1 mouse brain slices were evaluated using immunohistochemistry to confirm comparable Aβ pathology in both mouse models. H&E staining revealed typical amyloid morphology in the cortex of APP23 and APPPS1 mice but not in the cortex of control mice (Fig. S3, A).

IF staining showed a specific Aβ_1−42_ signal in the cortical regions of APP23 and APPPS1 mouse brain tissue but not in brain slices of age-matched controls (Fig. S3, B). APP23 revealed big, dense-core plaques, that were mainly distributed over the cortex, as well as CAA in the blood vessels. APPPS1 mice exhibited smaller, less dense-core plaques with a corona of diffuse amyloid, that were more distributed over the whole tissue section.

ICTAD-1 allows to differentiate Aβ_1−40_ from Aβ_1−42_ with different fluorescence emission [27]. In APP23 mice, green fluorescence emission showed Aβ_1−42_ in the inner of dense-core plaques in the brain parenchyma; and red fluorescence emission showed predominant Aβ_1−40_ pathology in CAA, as well as Aβ_1−40_ in the outer layer of parenchymal β-amyloid. In APPPS1 mice, the amyloid signal mainly consisted of the Aβ_1−42_-dominant parenchymal plaques in APPPS1 mice, surrounded by low Aβ_1−40_ (Fig. S4 A).

Quantification of the Aβ_1–40_/Aβ_1–42_ ratio in cortical PEA and CAA in 20.8-month-old APP23 and 15.8-month-old APPPS1 mice was assessed with ICTAD-1 by calculating the ratio of the fluorescence emission intensity of the red and green channel for each aggregate (PEA) and vessel (CAA) (Fig. 3, D). In APP23 mice, the Aβ_1–40_/Aβ_1–42_ ratio in PEA was 2.67 ± 0.89 (*n* = 1740 aggregates), and the Aβ_1–40_/Aβ_1–42_ ratio in CAA was 2.09 ± 0.83 (*n* = 247 vessels). In APPPS1 mice, the Aβ_1–40_/Aβ_1–42_ ratio in PEA was 0.89 ± 1.07 (*n* = 13217 aggregates), and the Aβ_1–40_/Aβ_1–42_ ratio in CAA was 1.10 ± 0.39 (*n* = 69 vessels). Only a low number of vessels in APPPS1 were CAA positive.

Further fluorescence evaluation with PIB confirmed the specific amyloid morphology, showing dense-core plaques and CAA in APP23 mice and more diffuse plaques in APPPS1 mice (Fig. S4, B, top row). FER staining revealed a strong fluorescence signal from the vessel wall in APP23 mice and a weaker signal from parenchymal amyloid plaques in APP23 and APPPS1 mice (Fig. S4, B, bottom row).

### In vitro autoradiography on mouse brain tissue

Autoradiography of [^3^H]FER revealed specific binding in cortical regions of APP23 mice, consistent with vascular amyloid distribution (Fig. 3A, top row). No significant binding was detected in APPPS1 and wild-type controls. In contrast, [^3^H]PIB showed strong labeling in the cortex of APP23 and APPPS1 mice (Fig. 3A, bottom row) with ~ 8-fold higher binding of [^3^H]PIB on APP23 mouse brain tissue (1637 ± 122 fmol/mg) compared to APPPS1 (210 ± 41 fmol/mg) and only background signal in control brain tissue. In contrast, [^3^H]FER binding was lower in both models, but ~ 80-fold selective to Aβ aggregates in the brain tissue of APP23 mice (82 ± 74 fmol/mg), with only background signal in APPPS1 and control brain tissue (Fig. 3, B).

(d_4_)-[^18^F]FER and [^11^C]PIB autoradiography confirmed this pattern, but showed slightly increased diffuse binding of (d_4_)-[^18^F]FER in APPPS1 mice but not in the negative control (Fig. 3, C), possibly due to a higher tracer concentration and partial parenchymal cross-reactivity.

Fluorescent colocalization with ICTAD-1 staining demonstrated overlap with (d_4_)-[^18^F]FER with Aβ_1−40_ (red) vascular deposits rather than Aβ_1−42_ predominant (green), parenchymal plaques (Fig. 3, D). These results supported CAA-preferential binding *in vitro.*

### In vivo pharmacokinetics and metabolic profile in healthy mice

**Fig. 4:**
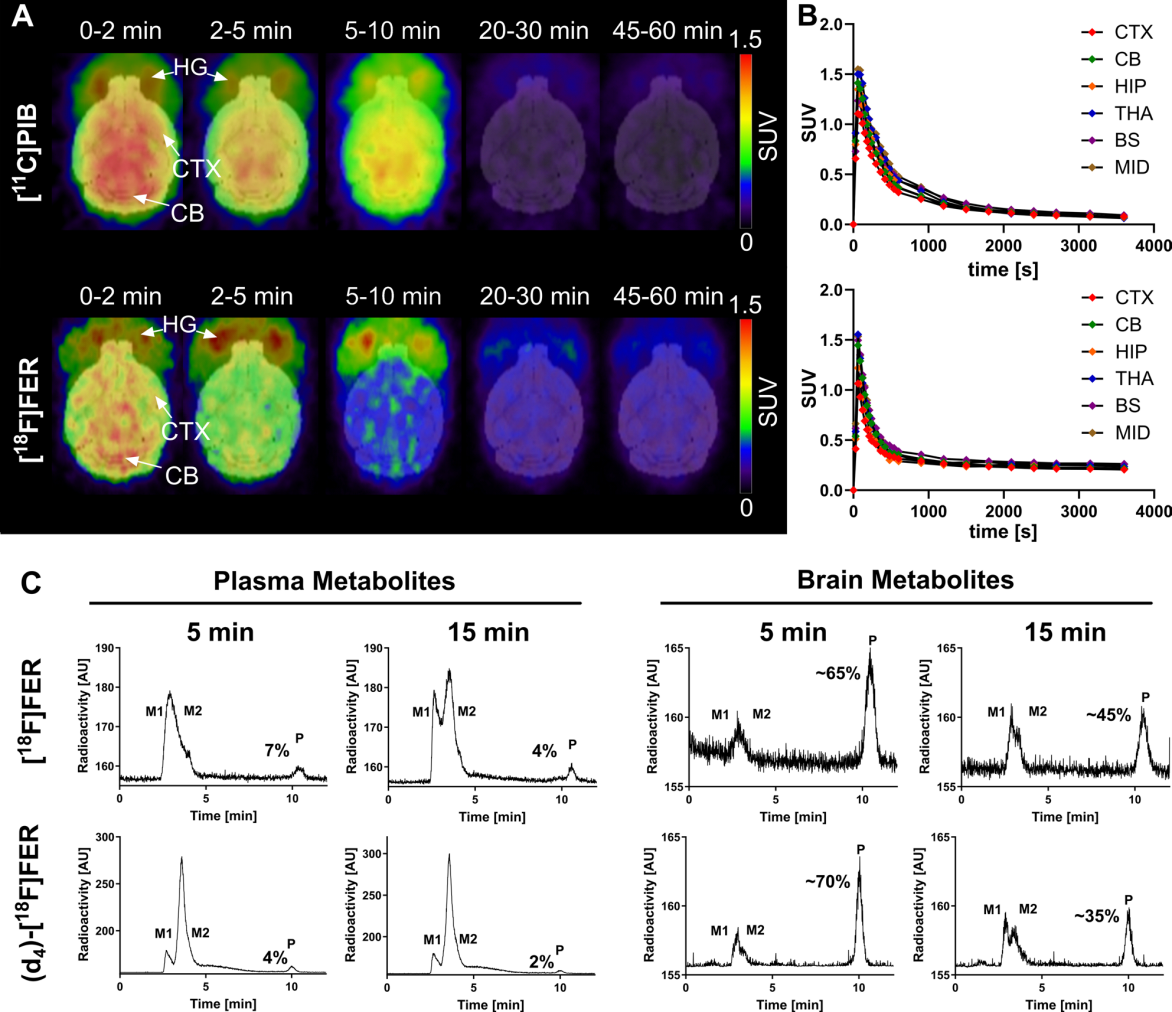
Pharmacokinetic profiles and metabolic stability of [^18^F]FER and comparison to [^11^C]PIB in vivo. (**A**) Representative dynamic PET images of [^11^C]PIB (top) and [^18^F]FER (bottom) in one mouse at multiple time points post injection. (**B**) Corresponding time activity curves (TACs) of selected brain regions showing initial uptake and clearance. (**C**) Metabolite analysis of [^18^F]FER and (d_4_)-[^18^F]FER in plasma and brain at 5 min and 15 min post-injection, indicating rapid metabolism and moderate brain stability. Abbreviations: FER, fluoroethylresorufin; PIB, Pittsburgh Compound B; PET positron emission tomography; SUV, standardized uptake value; HG, Harderian glands; CTX, cortex; CB, cerebellum; HIP, hippocampus, THA, thalamus; BS, brainstem; MID, midbrain; M1 and M2, metabolite 1 and 2; P, parent compound

In wild-type mice, PET scans of [^11^C]PIB showed rapid brain uptake and fast and complete washout from the brain (cortex t_1/2_ = 3.6 min) (Fig. 4, A top). [^18^F]FER showed a similar rapid brain uptake with peak SUVs ranging from 1.06 to 1.55 in different brain regions and a fast, but less complete washout from the brain (cortex t_1/2_ = 2 min) (Fig. 4, A, bottom), with cortical SUVs of ≤ 0.1 for [^11^C]PIB and ≥ 0.2 for [^18^F]FER after 60 min (Fig. 4, B).

Brain and plasma metabolite analysis of [^18^F]FER revealed one major polar plasma metabolite with 7% of the parent compound remaining in the plasma at five minutes after injection and two major polar plasma metabolites with 6% of the parent compound remaining at 15 min after injection.

Analysis of brain homogenates revealed one major brain metabolite with 70% and 50% of the parent compound present in the brain at five and 15 min after injections, respectively (Fig. 4, C).

To reduce metabolic degradation, a deuterated analog, (d_4_)-[^18^F]FER was synthesized (Fig. S5). Deuteration altered the metabolic profile, yielding a higher ratio of less polar metabolites, but did not significantly prolong metabolic stability (Fig. 4, C). Nevertheless, brain clearance improved with SUVs ≤ 0.2 at 60 min, compared to ≥ 0.3 for [^18^F]FER and cortex t_1/2_ = 2,75 min (Fig. S5, C), likely due to faster efflux of polar metabolites. These pharmacokinetic profiles motivated the use of (d_4_)-[^18^F]FER for subsequent in vivo imaging in transgenic animals.

### In vivo PET imaging in transgenic mouse models

**Fig. 5:**
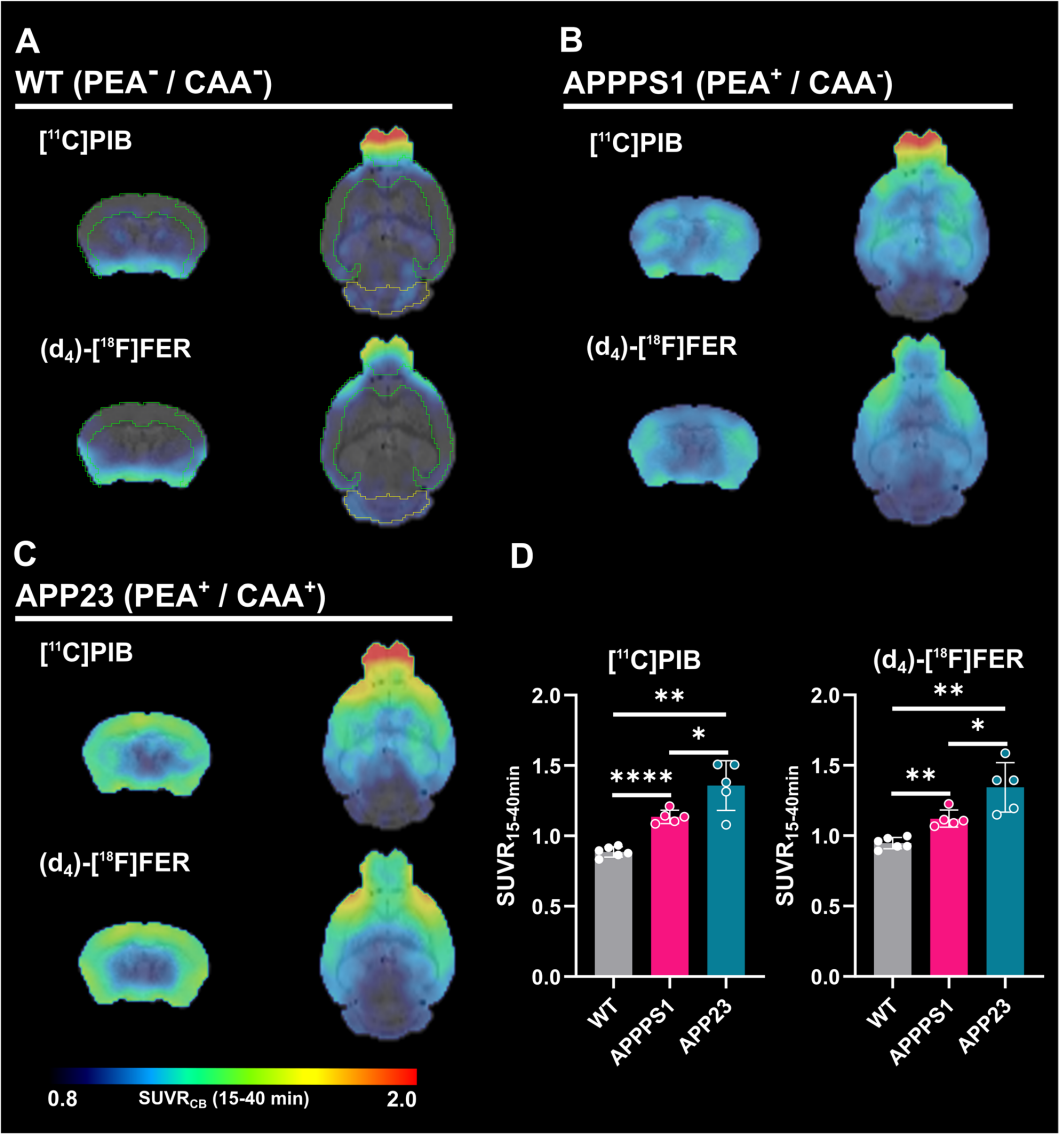
In vivo PET imaging and quantitative analysis of [^11^C]PIB and (d_4_)-[^18^F]FER in transgenic and wild-type mice. (**A**-**C**) Representative transversal (left), and coronal (right) mean SUVR images of [^11^C]PIB and (d_4_)-[^18^F]FER in (**A**) wild-type, (**B**) APPPS1, and (**C**) APP23 mice, using the cerebellum as reference region. (**D**) Quantification of mean cortical SUVRs values for (d_4_)-[^18^F]FER (left) and [^11^C]PIB (right) from the cortex of wild-type, APPPS1 and APP23 mice. CAA, cerebral amyloid angiopathy; PEA, parenchymal amyloidosis; WT, wild-type; PIB, Pittsburgh Compound B. FER, fluoroethylresorufin. SUVR, standardized uptake value ratio; CB, cerebellum

Dynamic PET imaging with [^11^C]PIB and (d_4_)-[^18^F]FER was performed in APP23 mice with predominant Aβ_1−40_ pathology, APPPS1 mice with predominant Aβ_1−42_ pathology, and control mice (Fig. 5A-C). Cortical SUV time activity curves showed a clear visual separation of transgenic animals from wild-type controls (Fig. S6, A and B). Cerebellar reference time activity curves did not show any visual separation with [C]PIB and a slightly separation with (d_4_)-[^18^F]FER in APPPS1 but not in APP23 (Fig. S6, C and D). Mean [^11^C]PIB SUV values show a significant difference from the wild-type in the cortex but not in the cerebellum, whereas mean (d_4_)-[^18^F]FER SUV values also show a significant difference from the wild-type in the cortex, but also in the cerebellum in APPPS1 mice (Fig. S6, E and F). Cortical SUVR time activity curves showed higher binding in APP23 mice for both [^11^C]PIB and (d_4_)-[^18^F]FER (Fig. S6, G and H). [^11^C]PIB demonstrated expected increased cortical retention in APPPS1 (SUVR = 1.14 ± 0.04) and APP23 mice (SUVR = 1.35 ± 0.15), but not in the wild-type (SUVR = 0.89 ± 0.03). A similar pattern was observed for (d_4_)-[^18^F]FER in APP23 compared to controls (SUVR = 1.34 ± 0.16 vs. 0.95 ± 0.04; *p* < 0.01) and in APPPS1 mice (SUVR = 1.12 ± 0.05; *p* < 0.05), as shown in Fig. 5, D. (d_4_)-[^18^F]FER and [^11^C]PIB SUVRs showed correlation along all groups (Fig. S6, I).

BP analysis with the simplified reference tissue model (SRTM) confirmed increased tracer binding in both transgenic strains compared to wild-type, but no complete separation between CAA- and PEA-dominant pathology (Fig. S6, J). Cortical retention patterns of (d_4_)-[^18^F]FER and [^11^C]PIB were spatially congruent, suggesting that in vivo, FER detects total fibrillar Aβ rather than vascular deposits exclusively.

## Discussion

This study provides a comprehensive preclinical evaluation of [^18^F]FER and its deuterated analog as potential PET tracer targeting vascular Aβ, the major neuropathological hallmark of CAA. The work spans molecular binding using amyloid fibrils, tissue autoradiography, pharmacokinetics, metabolism, and in vivo imaging in transgenic mice, allowing for an interpretation of specificity and selectivity. In addition, we directly compared FER with the established gold-standard Aβ tracer PIB. FER exhibited a high affinity for Aβ_1−40_ (K_d_ = 9.4 nM) with ~ 10-fold higher affinity binding than for Aβ_1−42_ (K_d_ = 89.1 nM), confirming the isoform preference previously suggested for resorufin derivatives. However, the number of binding sites was ~ 12-fold lower on Aβ_1−40_ fibrils than on Aβ_1−42_ fibrils, indicating that FER interacts with fewer, but higher-affinity binding sites on Aβ_1−40_ fibrils. Despite the higher affinity of FER for Aβ_1−40_, the comparable B_max_/K_d_ values (0.004 for Aβ_1−40_ and 0.005 for Aβ_1−42_) indicate that the overall binding capacity of both fibril types is similar. The affinities measured directly on Aβ fibrils were higher than the previously reported affinities obtained in Tg2576 mouse models of CAA, which may result from the different sensitivities of the applied methods [23, 26]. Although B_max_/K_d_ to synthetic Aβ_1−40_ and Aβ_1−42_ fibrils were similar, these experiments addressed only isoform preference and do not exclude compartment-specific binding mechanisms that may arise from structural or microenvironmental differences between vascular and parenchymal amyloid. The APP23 mouse model, used in the present study, has been previously described with extensive PEA, but also CAA with microhemorrhages, and Aβ_1−40_ dominating over Aβ_1−42_ with a ratio of 4.7:1 in 27–28 month-old mice [35–37]. The APPPS1 model has been described with Aβ_1−42_ dominating over Aβ_1−40_ with a ratio of 4.6:1 in 8-month old mice, and extensive PEA but minimal CAA [34]. For 16–22-month-old APPPS1 mice the total Aβ_1−40_/ Aβ_1−42_ was reported as 0.3:1 [35]. In the present study, both models showed a high load of parenchymal plaques, with large, non-homogeneously and clustered, dense-core plaques in APP23 mice and smaller, less dense-core plaques with a corona of diffuse β-amyloid in APPPS1 mice, that are homogeneously distributed without extensive clustering [38]. While absolute total Aβ_1−40_ and Aβ_1−42_ levels may vary with age, ICTAD-1 quantification in the present cohort confirms the expected divergence in fibrillar Aβ composition between APP23 (Aβ_1−40_/Aβ_1−42_ ratio 2.67 ± 0.89 in PEA and 2.09 ± 0.83 in CAA) and APPPS1 (Aβ_1−40_/Aβ_1−42_ ratio 0.89 ± 1.07 in PEA and 1.10 ± 0.39 in CAA) mice, which represents the relevant substrate for PET tracer binding. The observed ratios differed from previously reported literature values for total Aβ, which may be explained by several factors, including differences in animal age, the distinction between total Aβ and fibrillar Aβ quantified with ICTAD-1, and technical limitations of fluorescence-based quantification such as tissue autofluorescence and low signal intensities with high standard deviation. In addition, due to the configuration of the microscope system used in this study, excitation and emission were acquired in separate channels rather than using single-wavelength excitation with dual-emission detection as described in the original ICTAD-1 publication. These factors may have influenced the absolute ratio estimates, although the qualitative inter-model differences were consistent.

In autoradiography, [^3^H]FER and (d_4_)-[^18^F]FER showed strong labeling of APP23 brain sections but not in APPPS1 or wild-type controls. The autoradiographic signal pattern differed substantially from PIB, which labeled both vascular and parenchymal amyloid equally. Co-staining with ICTAD-1 fluorescence further confirmed that FER binding colocalized with Aβ_1−40_-enriched vascular deposits, while parenchymal Aβ_1−42_ contributed minimally. Notably, the NSB of FER was higher than the NSB of PIB, which might result from the higher lipophilicity of FER. In addition, we cannot rule out a contribution of erythrocytes or plasma protein binding to the overall binding signal.

In the present study, fibril binding assays and autoradiography were used as complementary approaches. While fibril assays allow determination of intrinsic affinity and species selectivity under controlled conditions, autoradiography assesses tracer binding in the context of complex brain tissue, where aggregate conformation, target accessibility, and non-specific binding may influence signal. The combination of both methods provides a stepwise evaluation from molecular interaction to tissue-level validation.

However, even this multi-level approach cannot fully predict in vivo tracer performance. Structural analyses of Aβ fibrils derived from human Alzheimer’s disease brain tissue have demonstrated that brain-derived aggregates may differ from synthetically generated fibrils and that distinct conformations can predominate between individuals [44, 45]. Moreover, pathological aggregates are subject to post-translational modifications and microenvironmental influences that are not recapitulated in simplified in vitro systems. Similar limitations have recently been emphasized in the broader field of PET tracer development for proteinopathies, highlighting that synthetic fibrils used for screening may not adequately reflect the structural and biochemical diversity encountered in vivo [46]. Together, these considerations underscore that tracer development requires a multi-level and iterative approach integrating radioligand binding assays, tissue-based tracer evaluation, and in vivo evaluation, as no single experimental tier alone is sufficient to reliably predict imaging performance.

Despite the promising in vitro finding, in vivo PET imaging revealed a different outcome. [^18^F]FER and (d_4_)-[^18^F]FER showed high brain uptake and fast brain washout in healthy mice, with pharmacokinetic profiles comparable to PIB. Tracer metabolism was relatively fast and was not improved after deuteration. However, (d_4_)-[^18^F]FER revealed improved washout kinetics and was therefore used for in vivo experiments in subsequent experiments in the transgenic mouse models. Tracer retention was observed in the cortex of transgenic APP23 mice and, to a lesser extent, in APPPS1 mice, resulting in cortical SUVRs that are similar to those of [^11^C]PIB. Although the cerebellum is widely used as a reference region in amyloid PET studies, low-level pathology at advanced ages cannot be fully excluded in transgenic models. In our study, cerebellar uptake was comparable between transgenic and wild-type animals for [^11^C]PIB, whereas (d_4_)-[^18^F]FER showed a modest increase in APPPS1 mice. As a result, inter-model SUVR differences reflect not only cortical tracer retention but also variability in the reference region. Notably, cortical SUV values did not differ significantly between APP23 and APPPS1 mice, indicating that reference region effects contribute to the observed SUVR differences. Nevertheless, transgenic animals remained clearly separable from wild-type controls. The overall retention pattern, therefore, reflected total fibrillar amyloid load rather than selective binding to vascular Aβ. Several pharmacokinetic parameters likely contributed to this lack of selectivity. First, the rapid and cerebral metabolism with only 45% of the parent compound remaining in the brain at 15 min post-injection, limiting the effective concentration of the parent tracer available for specific binding, and increases the contribution of the polar metabolites to the non-specific signal. Second, the relatively high lipophilicity of FER (clogP ~ 2.9) promotes plasma protein binding and nonspecific binding, further elevating the background signal. Third, the in vivo conditions differ substantially from static autoradiography, as regional blood flow and binding site accessibility may affect tracer binding. For example, in vitro equilibrium conditions favor detection of high-affinity binding, whereas in vivo, the tracer is exposed to transient binding opportunities during rapid perfusion. Since FER has a relatively fast washout rate, it may not achieve sufficient residence time to differentiate binding sites. These findings illustrate the typical trade-off in tracer design between achieving high affinity, sufficient brain penetration, and minimizing non-specific retention. While FER crossed the blood-brain-barrier effectively, this improvement in permeability comes at the cost of binding selectivity. Furthermore, the vascular amyloid load in mice is relatively small and distributed along fine leptomeningeal vessels, making spatial discrimination by small-animal PET difficult. In the APP23 model, which is PEA-dominant followed by strong CAA pathology, parenchymal plaques contain substantial amounts of Aβ_1−40_ surrounding Aβ_1−42_ cores. Thus, a tracer preferring Aβ_1−40_ will still bind to mixed deposits and cannot clearly distinguish vascular from parenchymal pathology.

Our observations align with and extend on prior reports on resorufin analogs and other vascular amyloid tracers. Lebouvier et al. described a strong affinity of resorufin for senile plaques, neurofibrillary tangles, and CAA in sections of AD patients [25], while Han et al. showed that resorufin-positive staining was occasionally present in the core of congophilic neuritic plaques in human AD brain tissue [23]. Abrahamson et al. confirmed in vivo labeling of both CAA and PEA in APPPS1 mice [24]. They also reported that resorufin labelled both plaques and CAA in tissue sections from the occipital cortex of an AD autopsy case and identified a more polar analog (“Compound 1”), which bound to vascular CAA after injection in vivo with limited access to parenchymal plaques due to limited blood-brain barrier penetration [24].

Interestingly, the lack of perfect selectivity does not preclude potential clinical utility. Other human studies with [^11^C]PIB and [^18^F]Florbetapir showed a differentiation of CAA patients from non-CAA subjects with intracerebral hemorrhage [47, 48]. Thus, even non-selective tracers may capture differences in regional amyloid distribution that correlate with vascular pathology. However, a CAA-selective tracer would be transformative, enabling early diagnosis, monitoring of anti-amyloid therapy, and risk stratification for ARIA.

## Conclusion

We conclude that (d_4_)-[¹⁸F]FER serves as a potential lead compound for the further development of a CAA-selective Aβ PET tracer, being limited by a lack of full selectivity and by high metabolite formation, but overcoming the initial limitations of resorufin. A next step towards a CAA selective Aβ PET tracer requires testing on diseased human brain tissue, as well as improvement of non-specific binding, selectivity, and metabolic stability. Despite these shortcomings, our results are valuable for guiding the rational design of next-generation tracers, and our findings provide an important reference point for ongoing CAA imaging probe development. The need for a truly CAA-selective PET tracer is greater than ever, given the increasing clinical relevance of CAA in the context of anti-amyloid therapies and ARIA risk management.

## Electronic Supplementary Material

Below is the link to the electronic supplementary material.

Additional file 1.

## Data Availability

The datasets generated and analyzed during the study are available from the corresponding author upon request.

## Abbreviations

Aβ: β-amyloid
ACN: Acetonitrile
AD: Alzheimer´s disease
A_m_: Molar activity
ARIA: Amyloid-related imaging abnormalities
ARIA-E: ARIA consistent of edema
ARIA-H: ARIA consistent of hemorrhages
BP: Binding potential
BSA: Bovine serum albumin
CAA: Cerebral amyloid angiopathy
clogP: Calculated logP
DAPI: 4′,6-Diamidin-2-phenylindol
DCM: Dichloromethane
DMF: Dimethylformamide
EOS: End of the synthesis
FER: Fluoroethylresorufin
FITC: Fluorescein isothiocyanate
FOV: Field of view
HPLC: High performance liquid chromatography
ICTAD-1: Imino-coumarin-thiazole for AD
IF: Immunofluorescence
LBD: Lewy body dementia
MRI: Magnetic resonance imaging
MS: Mass spectrometry
NGS: Normal goat serum
NSB: Non-specific binding
PDD: Parkinson´s disease dementia
PEA: Parenchymal Aβ deposits
PEG400: Polyethylene glycol
PET: Positron emission tomography
PIB: Pittsburgh compound B
PBS: Phosphate buffered saline
QMA: Quaternary methyl ammonium
RCP: Radiochemical purity
RCY: Radiochemical yield
SRTM: Simplified reference tissue model
SUV: Standardized uptake value
SUVR: Standardized uptake value ratio
TAC: Time activity curve
TB: Total binding
TBS: Tris-buffered saline
TBS-X: Tbs with Triton-X 100
TEM: Transmission electron microscopy
ThT: Thioflavin T
tPSA: Topological polar surface area
TRITC: Tetramethylrhodamine isothiocyanate
VOI: Volume of interest

## Declarations

### Use of AI

The manuscript underwent AI-assisted copy editing, such as correcting grammar and spelling, and ensuring consistency in style and format. This was performed with ChatGPT (OpenAI, San Francisco, CA, USA) and Grammarly (Grammarly Inc, San Francisco, CA, USA). The authors confirm that the edits reflect their original work and accept human accountability for the final version.
