# Supplementary material for: Preclinical evaluation of [18F]fluoroethylresorufin as PET tracer for cerebral amyloid angiopathy
Source: EJNMMI Res. 2026 Jul 16;16:110. doi: 10.1186/s13550-026-01470-4 (PMC13385527; doi:10.1186/s13550-026-01470-4)
Supplement: Supplementary file 1 — Additional file 1. [file 13550_2026_1470_MOESM1_ESM.pdf]

Supplementary Information

Preclinical Evaluation of [^{18}F]Fluoroethylresorufin as PET Tracer for Cerebral Amyloid Angiopathy

Daniel Bleher[1], Marilena Poxleitner[1], Ann-Kathrin Grotegerd[1], Oliver Hihn[1,2], Laura Kuebler[1,2], Gregory Bowden[1], Martin Schaller[3], Florian C. Maier[1], Bettina Weigelin[1,2], Bernd J. Pichler[1,2], Andreas Maurer[1,2] and Kristina Herfert[1]

[1]Werner Siemens Imaging Center, Department of Preclinical Imaging and Radiopharmacy, University of Tübingen, Tübingen, Germany

[2]Cluster of Excellence iFIT (EXC 2180) "Image Guided and Functionally Instructed Tumor Therapies", Eberhard Karls University, Tübingen, Germany

[3]Department of Dermatology, Eberhard Karls University, Tübingen, Germany

Corresponding author: Kristina Herfert

e-Mail: Kristina.herfert@med.uni-tuebingen.de

Precursor and standard synthesis of (d_4)-[^{18}F]FER

Synthesis of fluoroethylresorufin

The compound was synthesized as described (1, 2) with minor changes (Fig. S1, A). Resorufin (200 mg, 0.46 mmol) and potassium carbonate (140 mg, 138.2 mmol) were dissolved in 3 mL DMF. Fluoroethyl tosylate (240 mg, 0.55 mmol) was dissolved in 2 mL DMF and added to the reaction mix. The mixture was heated and stirred for 2h at 120°C under reflux cooling. After TLC indicated completion of the reaction, the solvent was then removed with a vacuum cold trap, and the crude product was then purified using flash preparative chromatography (gradient of MeOH in DCM) to afford the compound 7-(2-fluoroethoxy)-3*H*-phenoxazin-3-one as an orange solid (Yield 82%). LC/MS revealed a purity of 98.8%. MS: 260.1 $[M+H]^+$; 282.0 $[M+Na]^+$.

Synthesis of (d_4)-[^{18}F]FER precursor

The compound was synthesized as described(1) with minor changes (Fig. S1, B). Ethane-d_4-1,2-diol (250 mg, 3.79 mmol), DMAP (93 mg, 0.76 mmol), and triethylamine (1148 mg, 9.47 mmol) were dissolved in 40 mL DCM and cooled down to 0°C. Tosyl chloride (1799 mg, 9.47 mmol) was dissolved in 40 mL DCM and dropwise added to the reaction mix at 0°C. The mixture was stirred for 24 h at room temperature. After TLC indicated completion of the reaction, the solution was extracted with EtOAc three times. The combined organic layers were dried with anhydrous magnesium sulfate. After filtration, the solvent was removed by using arotary evaporator. The crude product residue was then purified using flash preparative chromatography (gradient of EtOAc in hexanes) to afford the compound ethane-1,2-diyl-d_4-bis(4-methylbenzenesulfonate) (Yield 82 %). MS: 375.0 $[M+H]^+$.

Sodium resorufin (200 mg, 0.85 mmol) and potassium carbonate (158 mg, 1.275 mmol) were dissolved in 5 mL DMF. Ethane-1,2-diyl- d_4-bis(4-methylbenzenesulfonate) (794.75 mg, 2.125 mmol) was dissolved in 2 mL DMF and slowly added to the reaction mix. The mixture was heated and stirred for 2h at 40°C under argon. TLC indicated the reaction was completed. After the reaction was completed, 50 mL of DCM was added. The organic phase was washed with 3x 50 mL H_2O and 3x 50 mL saturated NaCl solution. The organic layer was dried with anhydrous magnesium sulfate. After filtration, the solvent was removed by using a rotary evaporator. The crude product residue was then purified using flash preparative chromatography (gradient of EtOAc

in hexanes) to afford the (d_4)-[^{18}F]FER precursor 2-((3-oxo-3*H*-phenoxazin-7-yl)oxy)ethyl-1,1,2,2-d_4-4-methylbenzenesulfonate (Yield 79%). MS: 416.1, 417.1 $[M+H]^+$.

The [^{18}F]FER precursor (2-((3-oxo-3*H*-phenoxazin-7-yl)oxy)ethyl-4-methylbenzenesulfonate) was synthesized analog to the (d_4)-[^{18}F]FER precursor, starting with sodium resorufin and ethane-1,2-diyl bis(4-methylbenzenesulfonate).

Synthesis of ICTAD-1

The compound was synthesized as described in Yang et al.(3) with minor changes. 4-(Dimethylamino)phenylboronic acid (267 mg, 1.62 mmol) and 4-bromo-2-hydroxybenzaldehyde (250 mg, 1.25 mmol) were dissolved in 1,2-dimethoxyethane (35 mL). To the resulting solution, $Pd(PPh_3)_4$ (287 mg, 0.25 mmol) and an aqueous solution of sodium carbonate (2 M, 25 mL) were added under argon. The mixture was heated and stirred for 16 h at 80°C. After the reaction was completed, the solution was extracted with EtOAc three times. The combined organic layers were dried with anhydrous sodium sulfate. After filtration, the solvent was removed by rotary evaporator. The crude product residue was then purified using flash chromatography (gradient of EtOAc in hexanes) to afford the compound (4'-(dimethylamino)-3-hydroxy-[1,1'-biphenyl]-4-carbaldehyde) as a yellow crystalline solid. MS: 242.1, 243.1 $[M+H]^+$.
4'-(Dimethylamino)-3-hydroxy-[1,1'-biphenyl]-4-carbaldehyde (72 mg, 0.3 mmol) and 2- benzothiazoleacetonitrile (68 mg, 0.39 mmol) were dissolved in ethanol (3 mL) and dichloromethane (3 mL), followed by the addition of piperidine (2 drops). The resulting mixture was stirred for 3.5 h at room temperature. The final product was recrystallized thrice in a row in 50 ml pure EtOH at -78°C and then centrifuged at 5500g for 5 min. MS: 398.1, 399.1, 400.1 $[M+H]^+$; 199.6 $[M+2H]^{2+}$.

Supplemental figures

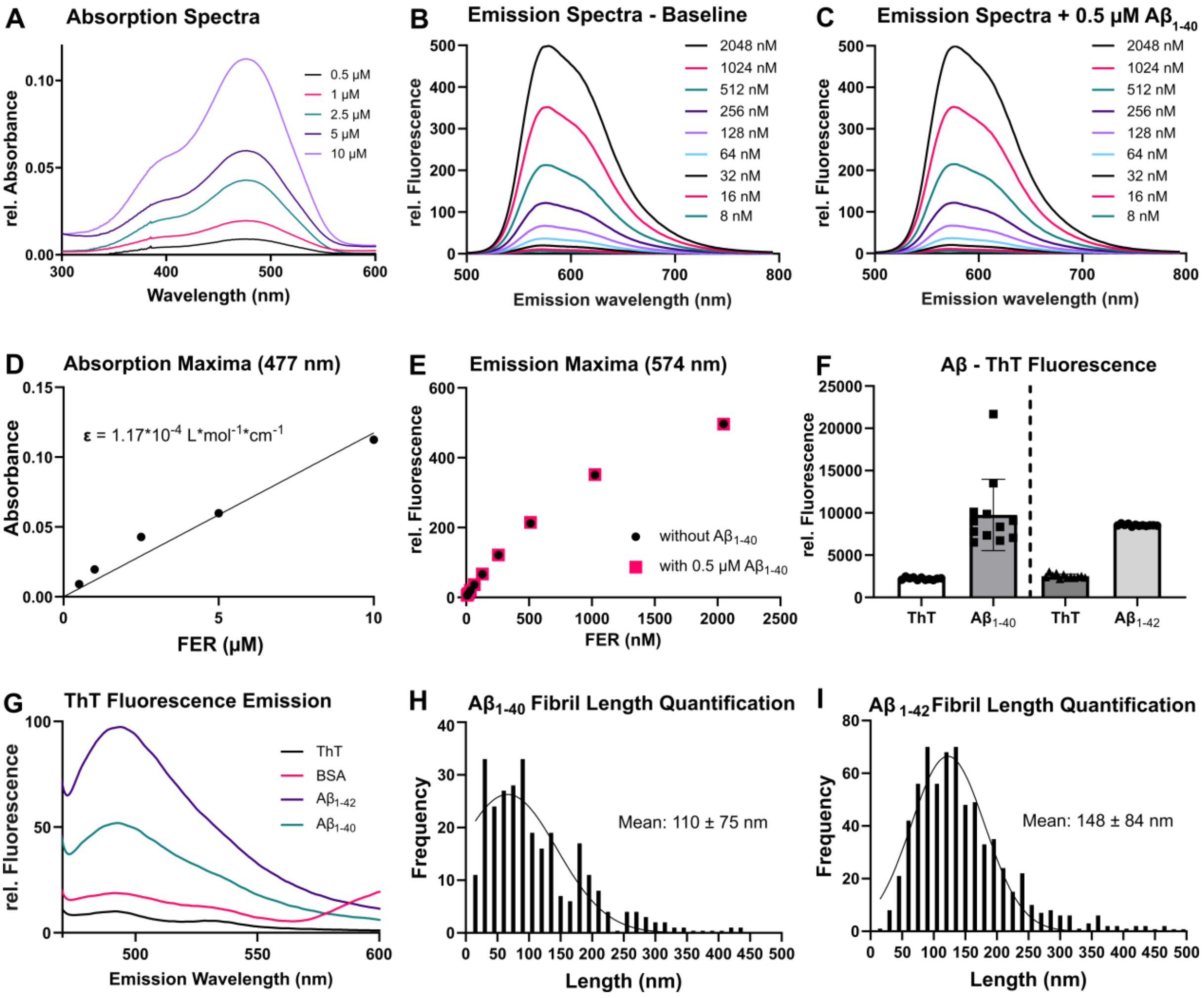

Figure S1: Spectroscopic characterization of FER and of synthetic human Aβ fibrils. (A) UV absorption spectra of FER at increasing concentrations. (B) Fluorescence emission spectra of FER at increasing concentrations (B; λ_{EX} = 477 nm). (C) Fluorescence emission spectra of FER at increasing concentrations in the presence of $A\beta_{1-40}$ fibrils (λ_{EX} = 477 nm). (D) Absorption maxima of FER at 477 nm. (E) Emission maxima of FER with and without added $A\beta_{1-40}$ fibrils (λ_{EX} = 477 nm). (F) Quantification of ThT fluorescence in presence of $A\beta_{1-40}$ and $A\beta_{1-42}$ fibrils. (G) ThT fluorescence emission spectra with added BSA, $A\beta_{1-40}$ and $A\beta_{1-42}$ fibrils (λ_{EX} = 450 nm). Fibril length distributions of (H) $A\beta_{1-40}$ and (I) $A\beta_{1-42}$ fibrils, respectively, with mean ± SD. FER, fluoroethylresorufin; ThT, Thioflavin T; BSA, bovine serum albumin.

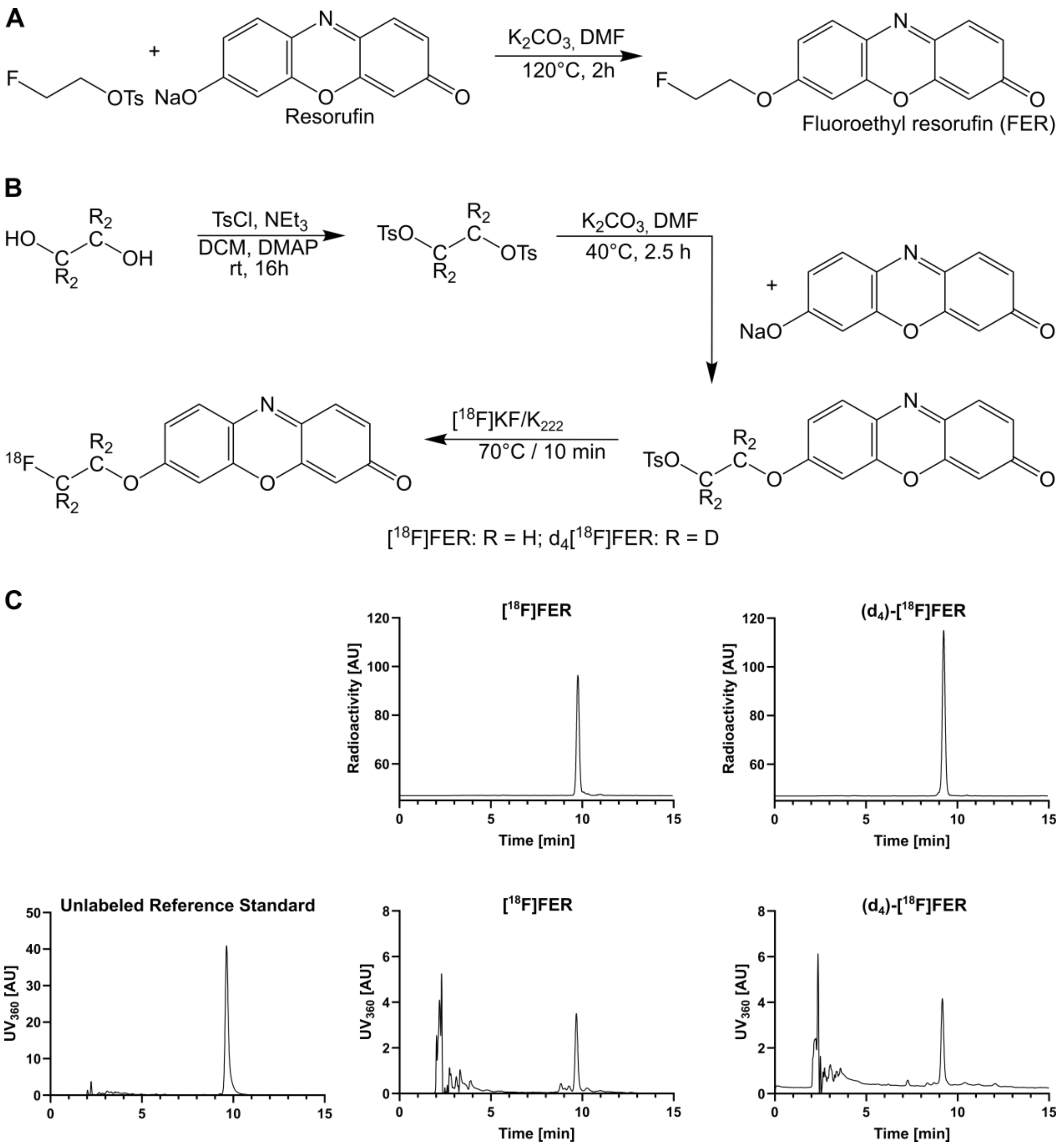

Figure S2: Synthesis of FER and its fluorine-18-labeled derivatives. (A) Chemical synthesis of FER by nucleophilic substitution of resorufin sodium salt with 2-fluoroethyl tosylate and K_2CO_3 in DMF at 120 °C for 2 h. (B) Preparation of [^{18}F]FER and (d_4)-[^{18}F]FER precursor via tosylation of ethylene glycol derivatives and subsequent coupling of resorufin. [^{18}F]FER and (d_4)-[^{18}F]FER radiosynthesis using [^{18}F]KF/Kryptofix 2.2.2. (C) Representative HPLC of the FER Reference (left), [^{18}F]FER (middle), and (d_4)- [^{18}F]FER (right) the radioactive channel (top) and at 360 nm (bottom). Chromatographic conditions: 48% MeCN in H_2O with 0.1% TFA; 1.5 mL/min. Abbreviations: FER, fluoroethylresorufin; HPLC, high-performance liquid chromatography; DMF, dimethylformamide; TFA, trifluoroacetic acid; MeCN, acetonitrile.

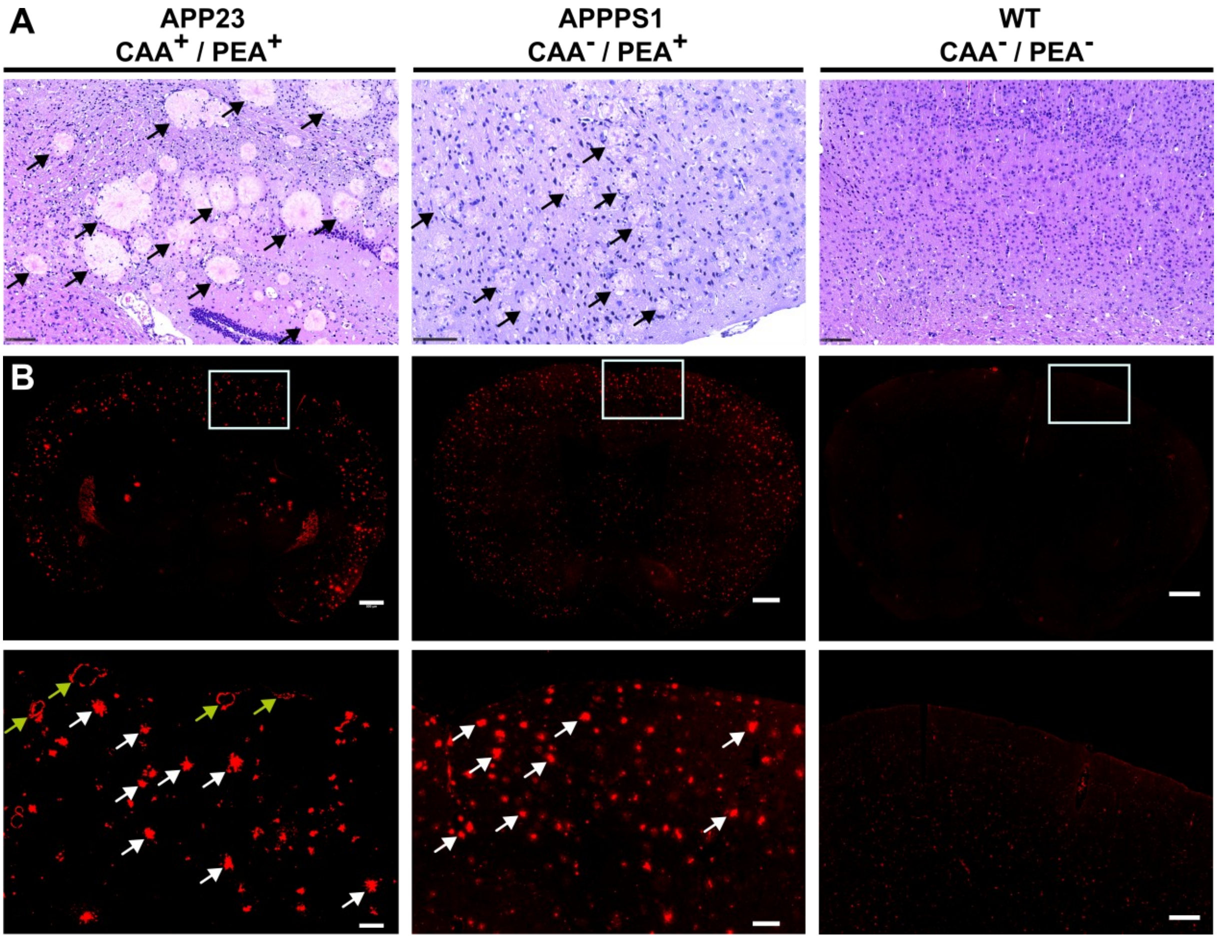

Figure S3: Histological characterization of amyloid pathology in transgenic mouse models. (A) Hematoxylin & Eosin (H&E) staining of cortical sections of APP23, APPPS1 and wild-type mice. (B) $A\beta_{1-42}$ antibody immunostaining of whole brain cortical sections (top) and 5x magnification of the upper cortex (bottom). APPP23: 21 months; APPPS1: 18.4 months. Black and white arrows, PEA; green arrows, CAA. Abbreviations: H&E, Hematoxylin and Eosin; CAA, cerebral amyloid angiopathy; PEA, parenchymal amyloidosis; WT, wild-type. Scale Bar (A) 100 µm; (B) 500 µm (top), 100 µm (bottom).

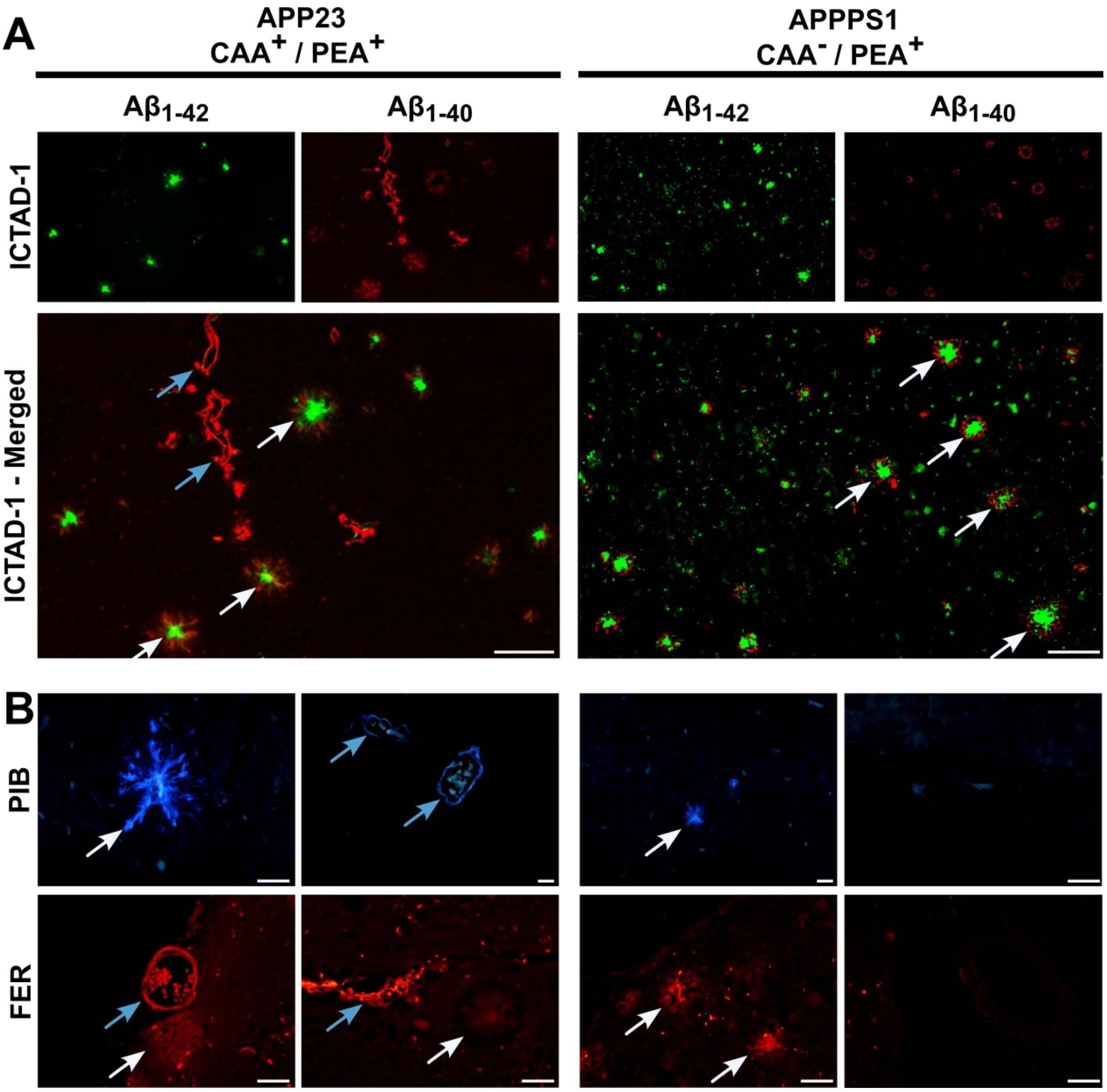

Figure S4: Comparative histochemical staining with ICTAD-1, PIB, and FER. (A) ICTAD-1 fluorescence staining showing selective visualization of vascular, parenchymal and cortical amyloid in APP23 and APPPS1 mice. Red channel: $A\beta_{1\text{-}40}$; green channel: $A\beta_{1\text{-}42}$; and merged image (bottom) illustrates spatial overlap. Blue arrows indicate vascular amyloid, white arrows indicate parenchymal amyloid deposits. The visualization of the merged images does not provide a quantitative representation of the Aβ ratio. (B) PIB (top) and FER (bottom) staining of cortical vessels and plaques in transgenic APP23 and APPPS1 mice. For APPPS1, left images show PEA, right images show vessels. Blue arrows, CAA; white arrows PEA. APPP23: 21 months; APPPS1: 18.4 months. Abbreviations: CAA, cerebral amyloid angiopathy; PEA, parenchymal amyloidosis; PIB, Pittsburgh Compound B; FER, fluoroethylresorufin. Scale Bar 100 µm.

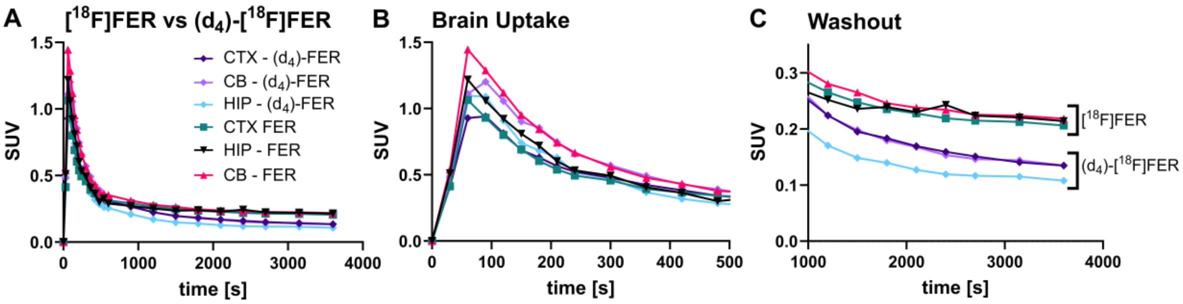

Figure S5: Brain pharmacokinetics of [^{18}F]FER and (d_4)-[^{18}F]FER. (A) Standardized uptake values **(**SUV) of [^{18}F]FER and (d_4)-[^{18}F]FER in cortex (CTX), cerebellum (CB), and hippocampus (HIP) following intravenous injection. (B, C) Zoomed time activity curves highlighting initial brain uptake (B) and washout (C). Abbreviations: FER, fluoroethylresorufin, CTX, cortex; CB, cerebellum, HIP, hippocampus, SUV, standardized uptake value.

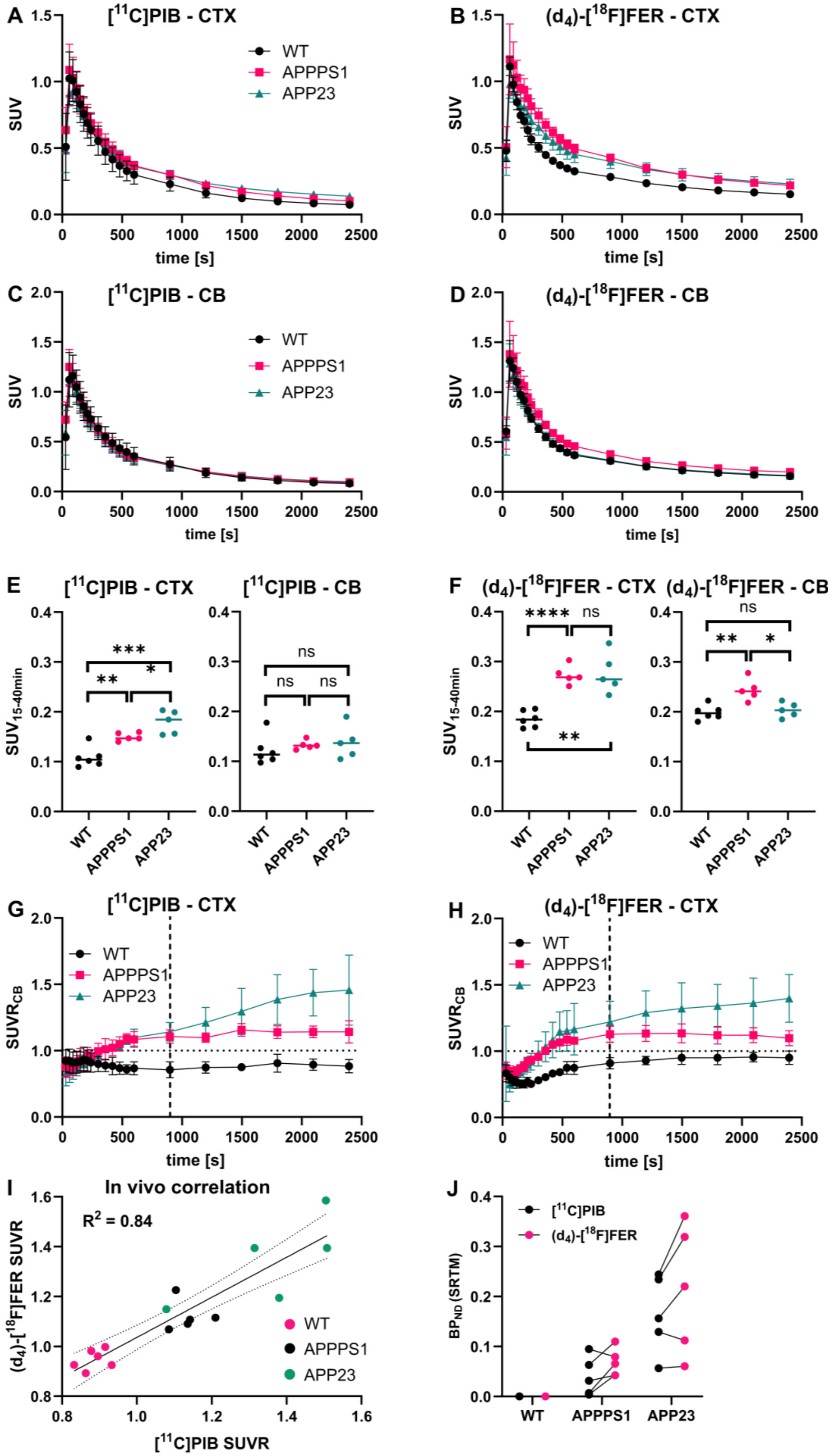
A
[11C]PIB - CTX
B
(d4)-[18F]FER - CTX
C
[11C]PIB - CB
D
(d4)-[18F]FER - CB
WT
APPPS1
APP23
SUV
time [s]
E
[11C]PIB - CTX
[11C]PIB - CB
F
(d4)-[18F]FER - CTX
(d4)-[18F]FER - CB
SUV15-40min
ns
G
[11C]PIB - CTX
H
(d4)-[18F]FER - CTX
SUVRCB
I
In vivo correlation
R2 = 0.84
(d4)-[18F]FER SUVR
[11C]PIB SUVR
J
[11C]PIB
(d4)-[18F]FER
BPND (SRTM)

Figure S6: Extended in vivo analysis of (d$_4$)-[^{18}F]FER and [^{11}C]PIB in transgenic mice. (A, B) Time-activity curves of [^{11}C]PIB and (d$_4$)-[^{18}F]FER in the cortex of WT, APPPS1 and APP23 mice. (C, D) Time-activity curves of [^{11}C]PIB and (d$_4$)-[^{18}F]FER in the cerebellum of WT, APPPS1 and APP23 mice. (E, F) Mean SUV values (15-40 min) of [^{11}C]PIB and (d$_4$)-[^{18}F]FER in the cortex and cerebellum of WT, APPPS1 and APP23 mice. (G, H) SUVR quantitative values of [^{11}C]PIB and (d$_4$)-[^{18}F]FER in the cortex of WT, APPPS1, and APP23 mice with cerebellum as reference region. (I) SUVR value (15-40 min) correlation across groups. (J) Binding potentials of [^{11}C]PIB and (d$_4$)-[^{18}F]FER in the different groups derived by simplified reference tissue modeling (SRTM). Abbreviations: FER, fluoroethylresorufin; PIB, Pittsburgh Compound B; CTX, cortex; SUV, standardized uptake value; WT, wild-type; SUVR, SUV ratios; BP, binding potential; SRTM, simplified reference tissue model.

References

1. Zipfel G, Han B, Mach R, Chu W, inventors; Washington University in St. Louis, assignee. Phenoxazine derivatives and methods of use thereof2015 2015/06/25.
2. Han BH, Zhou ML, Vellimana AK, Milner E, Kim DH, Greenberg JK, et al. Resorufin analogs preferentially bind cerebrovascular amyloid: potential use as imaging ligands for cerebral amyloid angiopathy. Mol Neurodegener. 2011;6:86.
3. Yang J, Zhu B, Yin W, Han Z, Zheng C, Wang P, et al. Differentiating Aβ40 and Aβ42 in amyloid plaques with a small molecule fluorescence probe. Chemical Science. 2020;11(20):5238-45.
